# Supplementary material for: Neutralization potency of monoclonal antibodies recognizing dominant and subdominant epitopes on SARS-CoV-2 Spike is impacted by the B.1.1.7 variant
Source: Immunity. 2021 Jun 8;54(6):1276–1289.e6. doi: 10.1016/j.immuni.2021.03.023 (PMC8015430; doi:10.1016/j.immuni.2021.03.023)
Supplement: Document S1. Figures S1–S5 and Table S1 [file mmc1.pdf]

Immunity, Volume *54*

Supplemental information

Neutralization potency of monoclonal antibodies recognizing dominant and subdominant epitopes on SARS-CoV-2 Spike is impacted by the B.1.1.7 variant

Carl Graham, Jeffrey Seow, Isabella Huettner, Hataf Khan, Neophytos Kouphou, Sam Acors, Helena Winstone, Suzanne Pickering, Rui Pedro Galao, Liane Dupont, Maria Jose Lista, Jose M. Jimenez-Guardeño, Adam G. Laing, Yin Wu, Magdalene Joseph, Luke Muir, Marit J. van Gils, Weng M. Ng, Helen M.E. Duyvesteyn, Yuguang Zhao, Thomas A. Bowden, Manu Shankar-Hari, Annachiara Rosa, Peter Cherepanov, Laura E. McCoy, Adrian C. Hayday, Stuart J.D. Neil, Michael H. Malim, and Katie J. Doores

Supplementary Figure S1: Donors used for B cell sorting have SARS-CoV-2 Spike reactive IgG+ B cells. Strategy to isolate SARS-CoV-2 Spike specific IgG$^+$ B cells. Example sorting for donor P008. Live CD3/CD8$^-$CD14$^-$CD19$^+$IgM$^-$IgD$^-$IgG$^+$Spike$^+$Spike$^+$ cells were sorted into individual wells. The heavy and light chains were reverse transcribed and amplified using nested PCR with gene specific primers. Related to **Figure 1**.

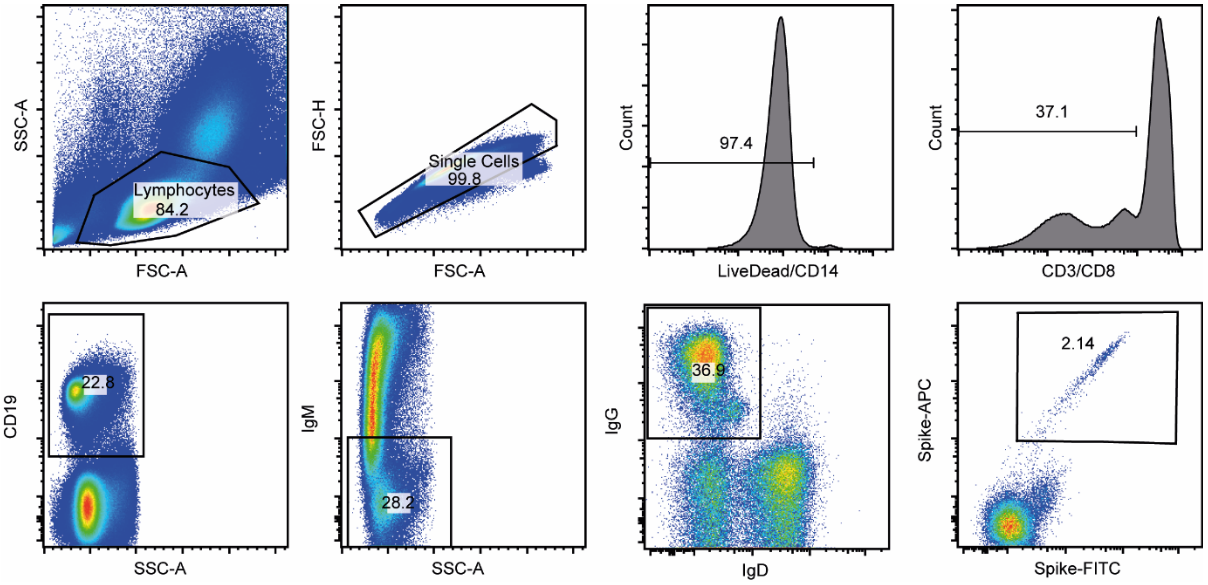

Supplementary Figure S2: SARS-CoV-2 specific mAbs have diverse gene usage. A) VH and VL germline gene usage for mAbs in the seven competition groups identified in **Figure 4A**. VH and VL genes are coloured according to the key. **B)** Pie charts showing percentage IGHV, IGHD, IGHJ, IGLV/IGLK and IGKJ/IGLJ usage for Spike reactive monoclonal antibodies. **C)** Bar graph showing the mean VH germline gene percentage usage in SARS-CoV-2 specific mAbs (blue) compared to a representative naïve repertoire (grey) (Briney et al., 2019). Error bars represent the standard deviation between donors used in the analysis (n = 3 for SARS-CoV-2 and n=10 for naïve repertoire). Differences between groups were determined using 2-way ANOVA with Šídák's multiple comparison test and *p* values <0.05 are shown. **D)** Single example of clonal expansion observed from P008. **E)** Sankey diagram showing the pairing between VH and VK or VL germline genes for SARS-CoV-2 mAbs. Related to **Figure 3**.

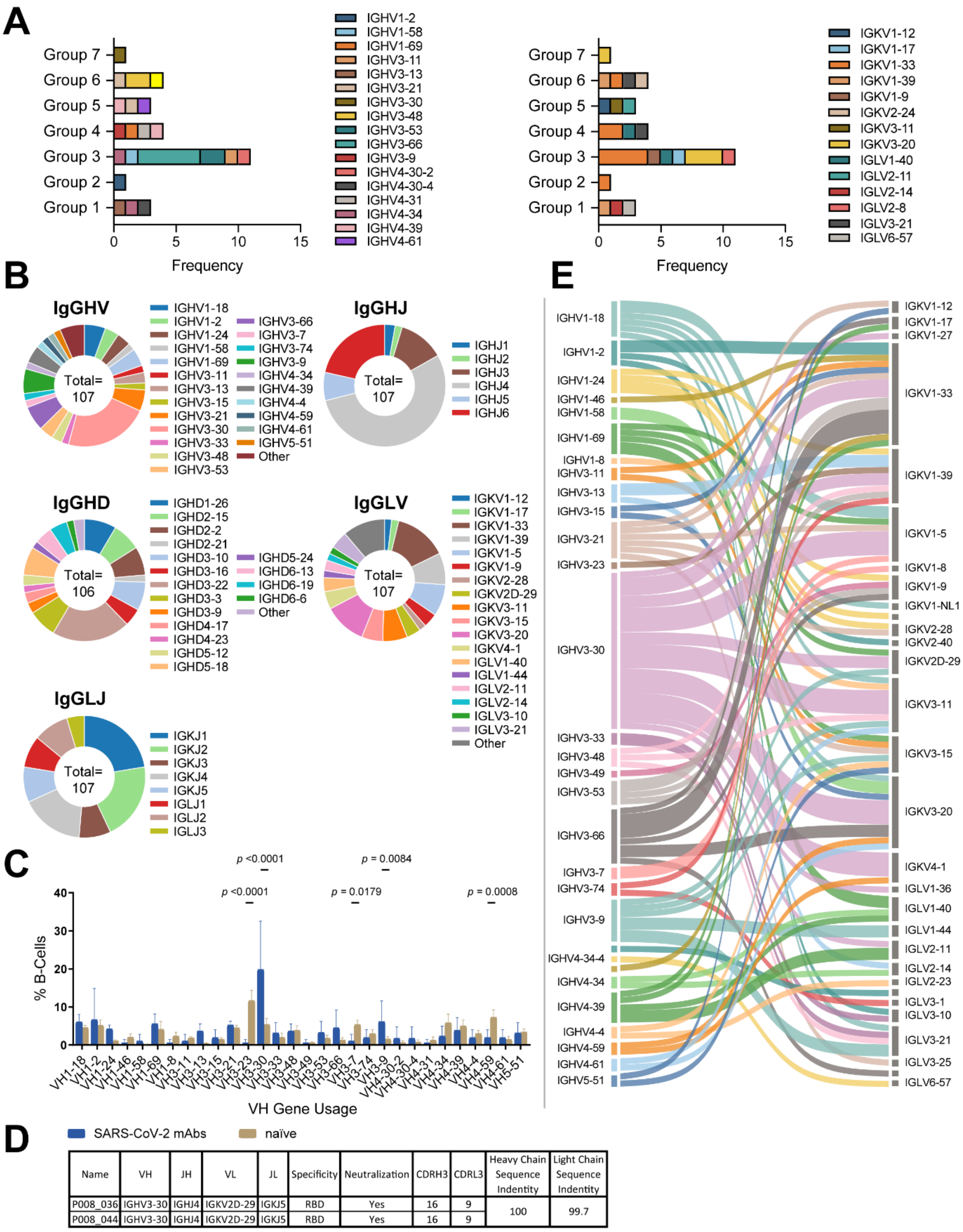

D

Name	VH	JH	VL	JL	Specificity	Neutralization	CDRH3	CDRL3	Heavy Chain Sequence Indentity	Light Chain Sequence Indentity
P008_036	IGHV3-30	IGHJ4	IGKV2D-29	IGKJ5	RBD	Yes	16	9	100	99.7
P008_044	IGHV3-30	IGHJ4	IGKV2D-29	IGKJ5	RBD	Yes	16	9		

Supplementary Figure S3: SARS-CoV-2 pseudovirus neutralization correlates and infectious virus neutralization and some nAbs cross-react with SARS-CoV. A) Correlation of mAb neutralization IC_{50} against infectious virus (y-axis) and pseudotyped virus (x-axis) (Spearman correlation, *r*. A linear regression was used to calculate the goodness of fit, r^2). **B)** Examples of shallow neutralization curves for Group 5 and Group 7 antibodies against SARS-CoV-2 pseudovirus. **C)** Group 6 nAbs show low neutralization plateaus against pseudovirus. **D)** Unlike the majority of other nAbs, Group 6 nAbs show 5-10 fold higher neutralization potency against infectious virus and reach neutralization plateaus of >95%. Binding of **E)** NTD and RBD nAbs and **F)** S2 non-neutralizing Abs to SARS-CoV-2 and SARS-CoV Spike proteins expressed on the surface of HEK 293T cells measured by flow cytometry. Also see, **Table S1**. Binding is reported as the % PE-positive cells. Antibodies are colour coded based on their competition group according to the key. **G)** Inhibition in binding of previously characterised SARS-CoV-2 nAbs (Brouwer et al., 2020) to Spike by $F(ab)_2$' fragments (generated through IdeS digestion of purified IgG) of representative members of each competition Group. Serial dilutions of $F(ab')_2$ (starting at 100-molar excess of the IC_{80} of Spike binding) were incubated for 1 hr before washing and addition of competing IgG (added at the IC_{80} of Spike binding). The percentage competition was calculated using the reduction in IgG binding in the presence of $F(ab')_2$ (at 100-molar excess of the IC_{80}) as a percentage of the maximum IgG binding in the absence of $F(ab')_2$. RBD, NTD and non-S1 specific nAbs were competed with the COVA mAbs specific for these domains only. Related **to Figure 4**.

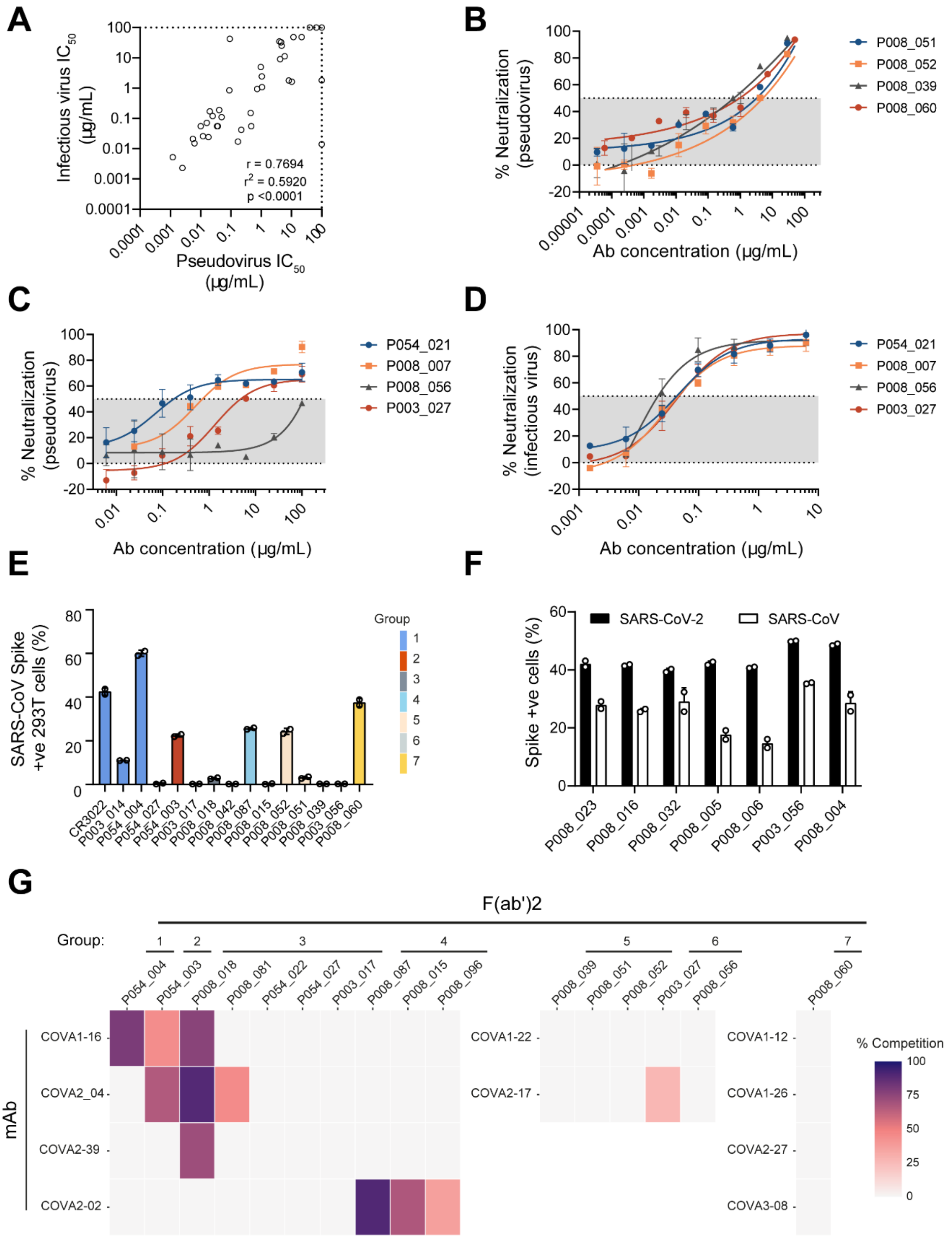

A
Infectious virus IC_{50} (μg/mL)
Pseudovirus IC_{50} (μg/mL)
r = 0.7694
r^2 = 0.5920
p <0.0001
B
% Neutralization (pseudovirus)
Ab concentration (μg/mL)
P008_051
P008_052
P008_039
P008_060
C
% Neutralization (pseudovirus)
Ab concentration (μg/mL)
P054_021
P008_007
P008_056
P003_027
D
% Neutralization (infectious virus)
Ab concentration (μg/mL)
P054_021
P008_007
P008_056
P003_027
E
SARS-CoV Spike +ve 293T cells (%)
Group
CR3022
P003_014
P054_004
P054_027
P054_003
P003_017
P008_018
P008_042
P008_087
P008_015
P008_052
P008_051
P008_039
P003_056
P008_060
F
Spike +ve cells (%)
SARS-CoV-2
SARS-CoV
P008_023
P008_016
P008_032
P008_005
P008_006
P003_056
P008_004
G
F(ab')2
Group:
mAb
P054_004
P054_003
P008_018
P008_081
P054_022
P054_027
P003_017
P008_087
P008_015
P008_096
P008_039
P008_051
P008_052
P003_027
P008_056
P008_060
COVA1-16
COVA2_04
COVA2-39
COVA2-02
COVA1-22
COVA2-17
COVA1-12
COVA1-26
COVA2-27
COVA3-08
% Competition

Supplementary Figure S4: Some nAbs show reduced neutralization potency against B.1.1.7 and related Spike variants. Neutralization of mAbs and plasma were tested against pseudoviruses expressing variant SARS-CoV-2 Spikes including D614G, N501Y, D614G ΔH69/V70 and D614G ΔY144 mutations and the B.1.1.7 and B.1.1.7+E484K variants. **A)** Change in neutralization IC_{50} (μg/mL) for D614G and N501Y mutation compared to wild-type Spike, D614G+ΔH69/V70, D614G+ΔY144 compared to D614G Spike, and B.1.1.7 and B.1.1.7+E484K compared to D614G Spike. nAbs are coloured by competition group according to the key. **B)** Neutralization curves for all nAbs tested. Graphs are arranged by competition group and are colour coded according to the key. Related to **Figure 6**

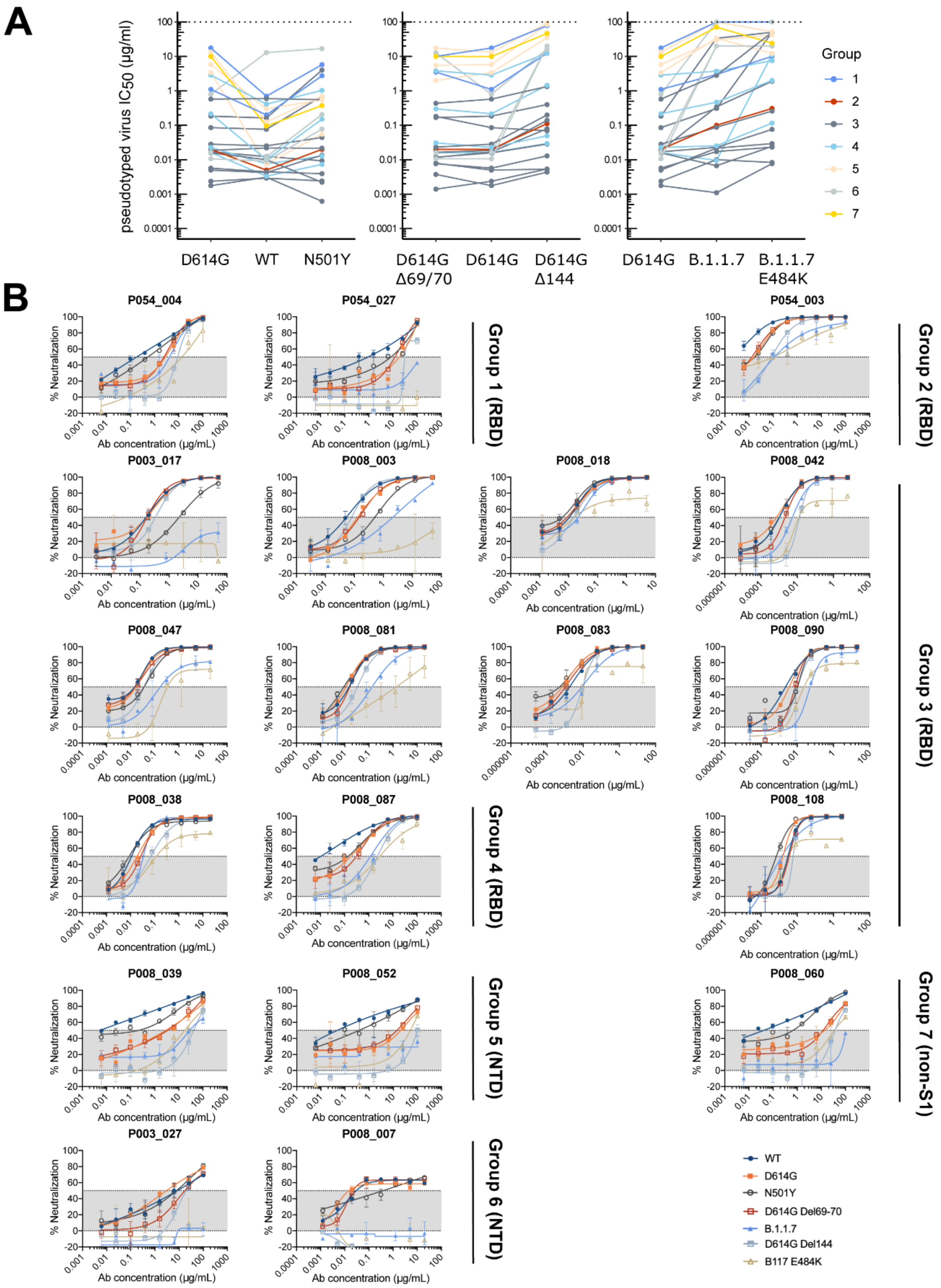

A
pseudotyped virus IC50 (µg/ml)
D614G
WT
N501Y
D614G Δ69/70
D614G
D614G Δ144
D614G
B.1.1.7
B.1.1.7 E484K
Group
1
2
3
4
5
6
7
B
P054_004
P054_027
P054_003
P003_017
P008_003
P008_018
P008_042
P008_047
P008_081
P008_083
P008_090
P008_038
P008_087
P008_108
P008_039
P008_052
P008_060
P003_027
P008_007
% Neutralization
Ab concentration (µg/mL)
Group 1 (RBD)
Group 2 (RBD)
Group 3 (RBD)
Group 4 (RBD)
Group 5 (NTD)
Group 6 (NTD)
Group 7 (non-S1)
WT
D614G
N501Y
D614G Del69-70
B.1.1.7
D614G Del144
B117 E484K

Figure S5: NTD-specific nAbs have the greatest reduction in neutralization potency against B.1.1.7 and related Spike variants. A) Fold changes in mAb IC_{50} compared to parent virus as indicated. **B)** Fold changes in plasma ID_{50} compared to parent virus as indicated. Each experiment was performed at least twice and the fold change is calculated from the average IC_{50} values. Related to **Figure 6** and **Figure S4**.

	Specificity	Group	From WT: D614G	From WT: N501Y	From D614G: D614G Del69/70	From D614G: D614G Del144	From D614G: B.1.1.7	From D614G: B.1.1.7 E484K	From B.1.1.7: B.1.1.7 E484K
P054_004	RBD	1	6	14	3	11	3	9	3
P054_027	RBD	1	25	8	-2	4	6	6	
P054_003	RBD	2	4	4	1	6	5	15	3
P003_017	RBD	3	1	25	-1	2	183	271	1
P008_003	RBD	3	1	9	2	-1	4	586	163
P008_018	RBD	3	1	-1	-1	5	1	4	3
P008_042	RBD	3	1	-1	1	2	3	4	1
P008_047	RBD	3	1	2	-1	7	3	9	3
P008_067	RBD	3	2	-5	-1	2	-2	2	4
P008_081	RBD	3	-1	-1	-1	7	18	102	6
P008_083	RBD	3	-1	-1	-2	2	4	4	-1
P008_090	RBD	3	1	3	2	1	4	6	1
P008_108	RBD	3	-2	-5	2	2	-2	4	7
P054_022	RBD	3	-1	-1	-1	2	5	87	18
P008_015	RBD	4	5	2	-1	2	-2	683	1166
P008_038	RBD	4	3	2	1	2	1	5	5
p008_087	RBD	4	22	15	1	7	2	9	4
P008_096	RBD	4	7	3	1	4	1	3	2
P008_039	NTD	5	450	7	-2	10	10	4	-3
P008_051	NTD	5	19	2	-1	15	5	7	1
P008_052	NTD	5	106	6	1	7	8	4	-2
P003_027	NTD	6	-16	1	16	20	125	125	
P008_007	NTD	6	1	17	-1	1216	1216	1216	
P054_021	NTD	6	1	8	1	1882	1882	1882	
P008_060	non-S1	7	105	4	1	5	7	2	-3

Specificity: RBD, NTD, non-S1

Group: 1, 2, 3, 4, 5, 6, 7

-fold loss neutralization

no difference

-fold increase neutralization

Base vius not neutralized

	From WT: D614G	From WT: N501Y	From D614G: D614G Del69/70	From D614G: D614G Del144	From D614G: B.1.1.7	From D614G: B.1.1.7 E484K	From B.1.1.7: B.1.1.7 E484K
P008	1	-1	1	2	8	n.d.	n.d.
P054	2	-2	-1	3	1	8	6

Table S1: SARS-CoV-2 mAbs target diverse epitopes. Gene usage, binding characteristics and neutralization properties of SARS-CoV-2 reactive mAbs expressed and purified on large scale. Antibodies are grouped by donor. Binding competition group for 27 mAbs is listed (see **Figure 4A**). EC_{50} was measured against Spike, RBD and NTD. For non-S1 binding mAbs, binding to S2 at 25 μg/mL was measured and + indicates binding. Neutralization ID_{50} was measured against infectious virus (with Vero-E6 target cells) and SARS-CoV-2 and SARS-CoV pseudovriuses (with HeLa-ACE2 target cells). The reported IC_{50} values are an average of three independent experiments. %ACE2 competition describes the ability of mAbs to prevent Spike binding to HeLa-ACE2 cells as measured by flow cytometry. The germline VH and VL usage is reported for each mAb. n.d., not determined. Related to **Figures 2, 3 and 4**.

mAb name	Specificty	Competition group	S EC50	RBD EC50	NTD EC50	S2 ELISA	SARS-CoV-2 WT FL IC50	SARS-CoV-2 WT PV IC50	SARS-CoV PV IC50	% ACE2 comp	VH	VL
P003_014	RBD	1	0.0076	0.0086	n.d.	n.d.	>100	>100	>50	86.3	IGHV3-13	IGKV1-39
P054_004	RBD	1	0.0360	0.0511	n.d.	n.d.	42.2300	0.2000	5.9585	95.2	IGHV4-34	IGLV2-14
P054_027	RBD	1	0.0077	0.0127	n.d.	n.d.	>100	0.7000	>50	92.9	IGHV4-30-4	IGLV6-57
P054_003	RBD	2	0.1756	0.2001	n.d.	n.d.	1.7900	0.0050	>100	98.5	IGHV1-2	IGKV1-33
P003_017	RBD	3	0.0057	0.0068	n.d.	n.d.	1.0800	0.1627	>100	96.5	IGHV3-66	IGKV1-9
P008_003	RBD	3	0.0756	0.0825	n.d.	n.d.	0.0170	0.0764	>50	99.7	IGHV4-34	IGLV1-40
P008_018	RBD	3	0.0714	0.0824	n.d.	n.d.	0.0550	0.0008	>50	99.9	IGHV3-66	IGKV3-20
P008_042	RBD	3	0.1277	0.1732	n.d.	n.d.	0.0240	0.0044	>50	99.9	IGHV3-66	IGKV3-20
P008_047	RBD	3	0.1731	0.2327	n.d.	n.d.	0.1893	0.0017	>50	99.7	IGHV3-53	IGKV1-33
P008_067	RBD	3	0.1638	0.1859	>20	n.d.	0.1700	0.0004	>50	99.1	IGHV3-66	IGKV1-17
P008_081	RBD	3	0.0384	0.0389	n.d.	n.d.	0.1159	0.0219	>50	99.5	IGHV1-58	IGKV3-20
P008_083	RBD	3	0.0203	0.1193	n.d.	n.d.	0.0260	0.0029	>50	99.9	IGHV3-66	IGKV1-33
P008_090	RBD	3	0.0182	0.0259	n.d.	n.d.	0.0709	0.0042	>50	99.9	IGHV3-53	IGKV1-33
P008_108	RBD	3	0.0092	0.0138	n.d.	n.d.	0.0023	0.0031	>50	100.0	IGHV3-11	IGKV1-33
P054_022	RBD	3	0.1741	0.1944	n.d.	n.d.	2.4400	0.5941	>50	98.5	IGHV4-30-2	IGLV2-8
P008_015	RBD	4	0.0337	0.0362	n.d.	n.d.	0.0553	0.0001	>50	80.2	IGHV3-9	IGLV3-21
P008_038	RBD	4	0.0578	0.0926	>10	n.d.	0.1100	0.0003	>50	42.3	IGHV1-69	IGLV1-40
P008_087	RBD	4	0.0212	0.0560	n.d.	n.d.	4.9600	0.0100	10.4600	48.5	IGHV4-31	IGKV1-33
P008_096	RBD	4	0.0299	0.0549	n.d.	n.d.	9.0920	0.4106	>50	50.6	IGHV4-39	IGKV1-33
P008_039	NTD	5	0.1014	>10	0.0240	n.d.	25.2800	0.0075	>50	91.2	IGHV3-21	IGKV1-12
P008_051	NTD	5	0.0914	>10	0.0561	n.d.	48.8200	0.3000	>50	74.5	IGHV4-61	IGKV3-11
P008_052	NTD	5	0.0853	>10	0.0625	n.d.	48.6500	0.1250	34.6400	80.2	IGHV4-39	IGLV2-11
P003_027	NTD	6	0.0039	>10	0.0065	n.d.	1.6400	12.9150	>50	47.4	IGHV3-48	IGKV1-39
P008_007	NTD	6	0.0700	>10	0.1710	n.d.	0.0549	0.0006	>100	66.3	IGHV3-48	IGLV3-21
P008_056	NTD	6	0.0602	>10	0.0488	n.d.	0.0136	>100	>50	36.8	IGHV3-21	IGKV1-33
P054_021	NTD	6	0.0263	>10	0.0643	n.d.	0.0421	0.0004	>100	51.0	IGHV1-24	IGKV2-24
P008_060	NON-S1	7	0.0311	>10	>10	n.d.	32.9000	0.0950	0.6434	50.7	IGHV3-30	IGKV3-20
P008_057	RBD	n.d.	0.0324	0.0597	>20	n.d.	0.0154	0.0063	>100	99.8	IGHV3-53	IGKV1-9
P008_070	RBD	n.d.	0.0342	0.1075	n.d.	n.d.	34.9500	4.0147	>100	47.3	IGHV3-30	IGKV1-33
P008_076	RBD	n.d.	0.0354	0.0384	n.d.	n.d.	0.8466	0.0889	0.4648	99.0	IGHV3-13	IGLV2-14
P008_086	RBD	n.d.	0.0338	0.0326	>20	n.d.	0.0052	0.0012	>100	99.3	IGHV1-58	IGKV3-20
P008_100	RBD	n.d.	0.0327	0.0364	>10	n.d.	11.3000	5.9766	>100	38.4	IGHV3-30	IGKV3-20
P008_103	RBD	n.d.	0.0663	0.1014	n.d.	n.d.	0.0215	0.0055	>100	99.8	IGHV3-66	IGKV1-33
P054_036	RBD	n.d.	0.0130	0.0293	>10	n.d.	0.0549	0.0100	>100	99.5	IGHV3-53	IGKV1-39
P054_050	RBD	n.d.	0.0268	0.0473	n.d.	n.d.	>100	66.6033	>100	60.1	IGHV3-13	IGKV1-39
P003_016	NTD	n.d.	0.1304	>20	2.0000	n.d.	0.1530	0.4494	>100	25.9	IGHV3-74	IGKV1-39
P003_055	NTD	n.d.	0.0600	>10	0.0546	n.d.	>100	>100	>100	n.d.	IGHV4-59	IGKV4-1
P008_001	NTD	n.d.	0.8157	>20	1.2000	n.d.	>100	>100	>100	37.3	IGHV3-49	IGKV1-9
P008_002	NTD	n.d.	0.0084	>20	0.0146	n.d.	>100	>100	>100	24.2	IGHV3-9	IGLV3-21
P008_014	NTD	n.d.	0.0662	>20	0.0215	n.d.	1.8120	>100	>100	43.0	IGHV3-15	IGKV1-33
P008_099	NTD	n.d.	0.0222	>20	0.1500	n.d.	>100	>100	>100	46.8	IGHV3-23	IGKV1-39
P054_044	NTD	n.d.	0.0308	>20	4.0000	n.d.	0.9428	0.7403	>100	46.7	IGHV1-69	IGKV1-5
P008_004	NON-S1	n.d.	0.0450	>10	>10	+	>100	>100	>100	61.4	IGHV3-7	IGKV1-5
P008_005	NON-S1	n.d.	0.2000	>10	>10	+	>100	>100	>100	22.8	IGHV3-30	IGKV1-5
P008_006	NON-S1	n.d.	0.1000	>10	>10	+	>100	>100	>100	16.0	IGHV3-30	IGKV1-5
P008_016	NON-S1	n.d.	0.1075	>10	>10	+	>100	>100	>100	39.9	IGHV4-59	IGKV3-20
P008_023	NON-S1	n.d.	0.4000	>10	>10	+	>100	>100	>100	22.7	IGHV3-11	IGKV3-15
P008_032	NON-S1	n.d.	0.0600	>10	>10	+	>100	>100	>100	23.3	IGHV3-30	IGKV3-11
