# Supplementary material for: Neutralization potency of monoclonal antibodies recognizing dominant and subdominant epitopes on SARS-CoV-2 Spike is impacted by the B.1.1.7 variant
Source: Immunity. 2021 Jun 8;54(6):1276–1289.e6. doi: 10.1016/j.immuni.2021.03.023 (PMC8015430; doi:10.1016/j.immuni.2021.03.023)
Supplement: Document S2. Article plus supplemental information [file mmc2.pdf]

Article

Immunity

Neutralization potency of monoclonal antibodies recognizing dominant and subdominant epitopes on SARS-CoV-2 Spike is impacted by the B.1.1.7 variant

Graphical abstract

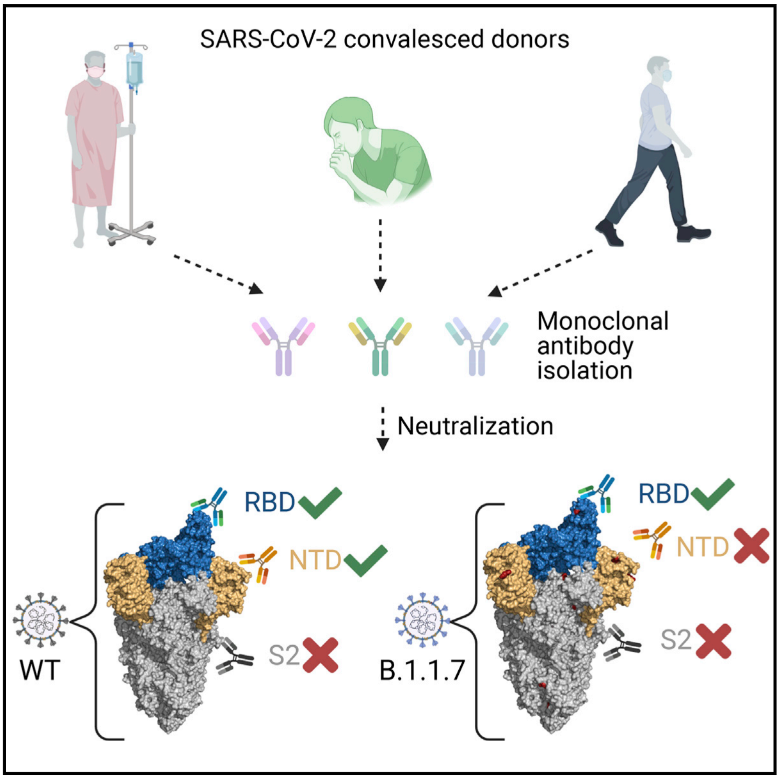

Highlights

- Potent nAbs were isolated from an asymptomatic donor with low plasma neutralization
- RBD-specific nAbs target epitopes overlapping with known RBD antibody classes
- NTD mutations in B.1.1.7 Spike confer neutralization resistance to NTD-specific nAbs
- Most RBD-specific nAbs retain potent neutralization of the B.1.1.7 variant

Authors

Carl Graham, Jeffrey Seow, Isabella Huettner, ..., Stuart J.D. Neil, Michael H. Malim, Katie J. Doores

Correspondence

katie.doores@kcl.ac.uk

In brief

The impact of mutations arising in SARS-CoV-2 Spike on antigenicity is still not known. Graham et al. isolate potent neutralizing monoclonal antibodies from individuals experiencing a range of COVID-19 disease severity that target RBD, NTD, and non-S1 epitopes. The B.1.1.7 variant of concern was most resistant to NTD-specific nAbs whereas RBD-specific nAbs retained potent neutralization.

Graham et al., 2021, Immunity *54*, 1276–1289
June 8, 2021 © 2021 The Authors. Published by Elsevier Inc.
https://doi.org/10.1016/j.immuni.2021.03.023

CellPress

Immunity

Article

Neutralization potency of monoclonal antibodies recognizing dominant and subdominant epitopes on SARS-CoV-2 Spike is impacted by the B.1.1.7 variant

Carl Graham,[1,8] Jeffrey Seow,[1,8] Isabella Huettner,[1] Hataf Khan,[1] Neophytos Kouphou,[1] Sam Acors,[1] Helena Winstone,[1] Suzanne Pickering,[1] Rui Pedro Galao,[1] Liane Dupont,[1] Maria Jose Lista,[1] Jose M. Jimenez-Guardeño,[1] Adam G. Laing,[2] Yin Wu,[2,3] Magdalene Joseph,[2,3] Luke Muir,[4] Marit J. van Gils,[5] Weng M. Ng,[6] Helen M.E. Duyvesteyn,[6] Yuguang Zhao,[6] Thomas A. Bowden,[6] Manu Shankar-Hari,[1] Annachiara Rosa,[3] Peter Cherepanov,[3] Laura E. McCoy,[4] Adrian C. Hayday,[2,3] Stuart J.D. Neil,[1,7] Michael H. Malim,[1,7] and Katie J. Doores[1,7,9,*]

[1]Department of Infectious Diseases, School of Immunology & Microbial Sciences, King's College London, London, UK
[2]Peter Gorer Department of Immunobiology, School of Immunology & Microbial Sciences, King's College London, London, UK
[3]The Francis Crick Institute, UK
[4]Division of Infection and Immunity, University College London, London, UK
[5]Department of Medical Microbiology, Academic Medical Center, University of Amsterdam, Amsterdam Institute for Infection and Immunity, Netherlands
[6]Division of Structural Biology, Wellcome Centre for Human Genetics, University of Oxford, Oxford, UK
[7]Genotype-to-Phenotype UK National Virology Consortium
[8]These authors contributed equally
[9]Lead Contact
*Correspondence: katie.doores@kcl.ac.uk
https://doi.org/10.1016/j.immuni.2021.03.023

SUMMARY

Interaction of the SARS-CoV-2 Spike receptor binding domain (RBD) with the receptor ACE2 on host cells is essential for viral entry. RBD is the dominant target for neutralizing antibodies, and several neutralizing epitopes on RBD have been molecularly characterized. Analysis of circulating SARS-CoV-2 variants has revealed mutations arising in the RBD, N-terminal domain (NTD) and S2 subunits of Spike. To understand how these mutations affect Spike antigenicity, we isolated and characterized >100 monoclonal antibodies targeting epitopes on RBD, NTD, and S2 from SARS-CoV-2-infected individuals. Approximately 45% showed neutralizing activity, of which ~20% were NTD specific. NTD-specific antibodies formed two distinct groups: the first was highly potent against infectious virus, whereas the second was less potent and displayed glycan-dependant neutralization activity. Mutations present in B.1.1.7 Spike frequently conferred neutralization resistance to NTD-specific antibodies. This work demonstrates that neutralizing antibodies targeting subdominant epitopes should be considered when investigating antigenic drift in emerging variants.

INTRODUCTION

Severe acute respiratory syndrome coronavirus 2 (SARS-CoV-2) is the causative agent of COVID-19. SARS-CoV-2 belongs to the *Betacoronavirus* genus of the *Coronaviridae* family, alongside severe acute respiratory syndrome coronavirus (SARS-CoV) and Middle East respiratory syndrome coronavirus (MERS-CoV) (Lu et al., 2020). The positive sense RNA genome encodes four structural proteins; Spike (S), envelope (E), membrane (M), and nucleocapsid (N) (Jiang et al., 2020). Spike is responsible for interaction with the human angiotensin-converting enzyme 2 (ACE2) receptor and subsequent virus-cell membrane fusion and thus is the key target for neutralizing antibodies (nAbs) (Pinto et al., 2020). The Spike glycoprotein assembles into homotrimers on the viral membrane, with each Spike monomer encompassing two functional subunits, S1 and S2. The S1 subunit contains the N-terminal domain (NTD) and the receptor binding domain (RBD). The RBD encompasses the receptor binding motif (RBM) that directly contacts the ACE2 receptor. The S2 subunit, containing the fusion peptide, two heptad repeats (HR1 and HR2), the cytoplasmic tail and the transmembrane domain, is crucial for viral membrane fusion (Yao et al., 2020).

Despite the recent emergence of SARS-CoV-2 in the human population, a rapid understanding of the antibody response arising from infection has emerged (Beaudoin-Bussières et al., 2020; Crawford et al., 2020; Dan et al., 2021; Muecksch et al., 2020; Okba et al., 2020; Pickering et al., 2020; Prévost et al., 2020; Seow et al., 2020). The majority of SARS-CoV-2 infected individuals have been shown to generate an antibody response 5–15 days post onset of symptoms (POS) that peaks after ~3–4 weeks and then starts to decline (Crawford et al., 2020; Muecksch et al., 2020; Prévost et al., 2020; Seow et al., 2020).

 © 2021 The Authors. Published by Elsevier Inc.
This is an open access article under the CC BY license (http://creativecommons.org/licenses/by/4.0/).

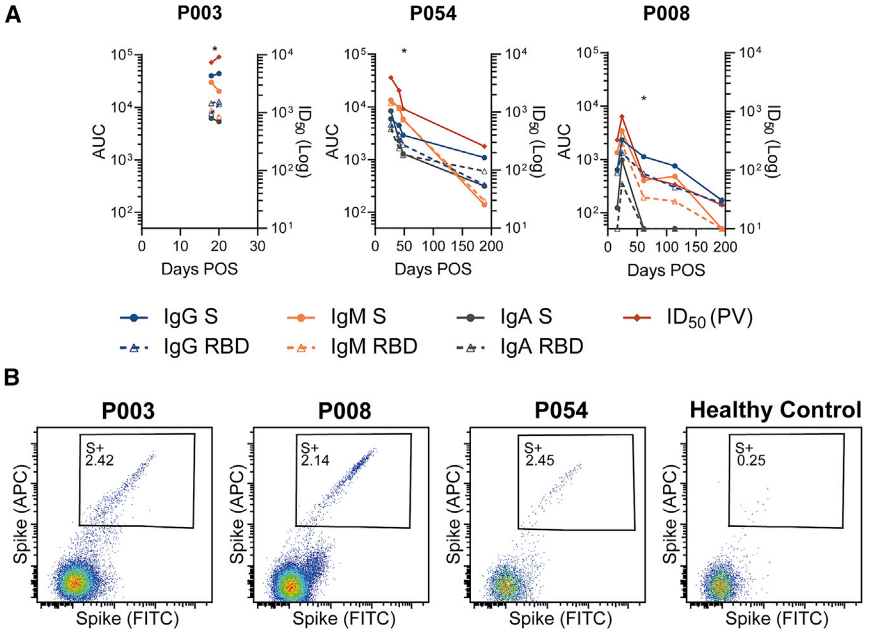

Figure 1. Donors used for B cell sorting have Spike binding IgG, IgA and IgM, nAbs and SARS-CoV-2 Spike reactive IgG+ B cells

(A) Kinetics of the antibody binding response (IgM, IgA, IgG against S and RBD) and neutralization activity against SARS-CoV-2 pseudovirus (PV) for donors P003, P008, and P054 in the acute and convalescent phase. ELISA data is reported as area under the curve (AUC, left y axis). Neutralization ID_{50} is shown on the right y axis. The asterisk indicates the time point from which mAbs were cloned for each donor. Experiments were performed in duplicate and repeated twice where plasma was available.

(B) Fluorescent activated cell sorting (FACS) showing percentage of $CD19^{+}IgG^{+}$ B Cells binding to SARS-CoV-2 Spike. A healthy control PBMC sample collected prior to the COVID-19 pandemic was used to measure background binding to Spike. The full gating strategy can be found in Figure S1.

The magnitude of the nAb response, which is thought to be important for protection from re-infection and/or disease, has been associated with disease severity. Specifically, those with most severe disease typically develop the strongest antibody response, whereas those experiencing mild disease, or who are asymptomatic, can have lower levels of neutralizing activity detectable in their sera (Guthmiller et al., 2021; Laing et al., 2020; Legros et al., 2021; Rees-Spear et al., 2021; Seow et al., 2020; Zohar et al., 2020). Antibodies targeting RBD have been suggested to account for >90% of neutralizing activity in convalescent sera (Greaney et al., 2021; Piccoli et al., 2020), and several neutralizing epitopes on RBD that are targeted by highly potent monoclonal antibodies (mAbs) have been molecularly characterized (Barnes et al., 2020; Brouwer et al., 2020; Piccoli et al., 2020; Pinto et al., 2020; Rogers et al., 2020; Tortorici et al., 2020; Wang et al., 2020; Wu et al., 2020b). Reports suggest that escape from RBD-mediated neutralization is occurring in variant strains that are emerging globally, which include mutations within RBD that have been postulated to enable escape (Muecksch et al., 2020; Starr et al., 2021; Wibmer et al., 2021). This highlights the need to identify nAbs that bind epitopes outside RBD and to understand the role these nAbs play in protection from re-infection or following vaccination. Identification of neutralizing epitopes beyond RBD is therefore important for the development of synergistic nAb cocktails for immunotherapy and passive vaccination, and it will also be critical for evaluating the relevance of potential immune escape viral variants as they arise, for example, the recently identified B.1.1.7 (Rambaut et al., 2020). We therefore sought to isolate SARS-CoV-2 nAbs from three donors experiencing either severe, mild, or asymptomatic COVID-19 using an un-cleaved, pre-fusion stabilized trimeric Spike glycoprotein (Wrapp et al., 2020) as antigen bait to further characterize the neutralizing epitopes present on SARS-CoV-2 Spike.

Here, we isolated 107 mAbs across three donors, of which 47 (43.9%) showed neutralizing activity. The majority (72.3%, 34/47) of the nAbs targeted RBD and formed four distinct competition groups. 21.3% (10/47) of nAbs targeted the NTD and formed two separate groups. One NTD group contained potent nAbs able to neutralize infectious virus at a similar potency to RBD-targeted nAbs. The second NTD group, although less potent, showed glycan-dependent neutralization activity. NTD-specific nAbs showed a substantial decrease in neutralization potency against the recently reported highly transmissible B.1.1.7 variant of concern, whereas RBD-specific nAbs were either unaffected or showed lower decreases in potency, indicating that nAbs against epitopes outside RBD are important to consider when investigating antigenic drift surveillance and identifying newly emerging SARS-CoV-2 variants of concern.

RESULTS

nAb responses following SARS-CoV-2 infection differ between donors with varied COVID-19 symptoms

We have previously studied antibody responses following SARS-CoV-2 infection (Laing et al., 2020; Seow et al., 2020). To study the widest range of SARS-CoV-2 nAbs at the monoclonal level, we selected three donors experiencing a range of COVID-19 severity (Characterization and Management of, 2020). P003 was hospitalized and spent time in ICU, P054 was symptomatic but did not require hospitalization, and P008 was asymptomatic and SARS-CoV-2 infection was only identified through serology screening (Laing et al., 2020). Longitudinal plasma samples were used to measure binding and neutralization titers (Figure 1A). As we and others have previously shown (Guthmiller et al., 2021; Laing et al., 2020; Legros et al., 2021; Rees-Spear et al., 2021; Seow et al., 2020; Zohar et al., 2020), the highest neutralization titer was detected in the individual with most severe disease (ID_{50} 9,181) and the lowest neutralization titer in the asymptomatic individual (ID_{50} 820). The nAb response declined during the convalescent period with neutralizing ID_{50} values reduced to

258 in P054 and 25 in P008 after 188 and 194 days post-onset of symptoms respectively. Plasma IgG, IgM and IgA binding to Spike and RBD were also measured using ELISA, and although IgG to Spike and RBD remained detectable, a large decrease from peak binding was observed (Figure 1A). These results confirm previous observations that disease severity is associated with the magnitude of the nAb response (Seow et al., 2020).

Antibodies generated following SARS-CoV-2 infection target RBD and other epitopes on S1 and Spike

Next, we used antigen-specific B cell sorting to isolate mAbs specific for SARS-CoV-2 Spike. PBMCs were available for sorting at days 20, 48, 61 POS from donors P003, P054, and P008, respectively. We used an uncleaved Spike that was stabilized in the prefusion conformation (GGGG substitution at furin cleavage site and 2P mutation [Wrapp et al., 2020]) as sorting bait to allow isolation and characterization of mAbs against the full range of neutralizing and non-neutralizing epitopes. 2.39%, 2.14%, and 2.45% of CD19+IgG+ B cells bound to Spike in donors P003, P008, and P054, respectively, compared to 0.25% for a pre-COVID-19 healthy control sample (Figure 1B and Figure S1). Despite a low ID_{50} of 76 for P008 at day 61 POS, Spike-reactive IgG^+ B cells were detected at a similar frequency to P054 with an ID_{50} of 1,144. 150 Spike-reactive cells were sorted from P003 and P008 and 120 cells from P054. The heavy and light chains were reverse transcribed and amplified using nested PCR with gene specific primers. The purified PCR products were ligated into heavy and light chain expression vectors using Gibson assembly, and the ligation products directly transfected into 293T cells (Rogers et al., 2020). Spike-reactive mAbs were identified by measuring Spike binding and IgG expression of supernatants using ELISA. The transformed Gibson products of Spike reactive mAbs were sequenced and used for gene analysis (see Figure 3 and Figure S2). Small-scale expression of sequenced antibodies was used to determine specificity toward Spike, S1, NTD, and RBD in ELISA (Figure 2A). In total, 107 Spike-reactive mAbs were identified and sequenced, 24, 19, and 64 from donors P003, P054, and P008, respectively (Figure 2B). 38/107 (35.5%) of the Spike reactive mAbs were RBD-specific, 35/107 (32.7%) were NTD specific, and 1/107 (0.9%) bound S1 only (Figure 2C). 33/107 (30.8%) mAbs only bound Spike, suggesting these mAbs are either specific for S2 or bind quaternary epitopes that span multiple subunits (Liu et al., 2020b). The distribution of mAb epitopes targeted differed between donors, with P003 mAbs predominantly binding non-S1 epitopes (Figure 2D).

SARS-CoV-2 neutralization activity of small-scale purified mAb or concentrated supernatant was determined using an HIV-1 (human immunodeficiency virus type-1)-based virus particles, pseudotyped with SARS-CoV-2 Spike and a HeLa cell-line stably expressing the ACE2 receptor (Seow et al., 2020). 47/107 (43.9%) of cloned mAbs had neutralizing activity, an observation which highlights the presence of exposed but non-neutralizing epitopes on Spike, including RBD, that generate a strong antibody response. 34/37 (91.9%) of RBD-specific mAbs were neutralizing, whereas only 10/35 (28.6%) of NTD-specific and 3/34 (8.8%) of the non-S1 binding mAbs had neutralizing activity (Figure 2C). Therefore, RBD was the dominant target for the nAbs isolated in this study consistent with prior literature (Piccoli et al., 2020).

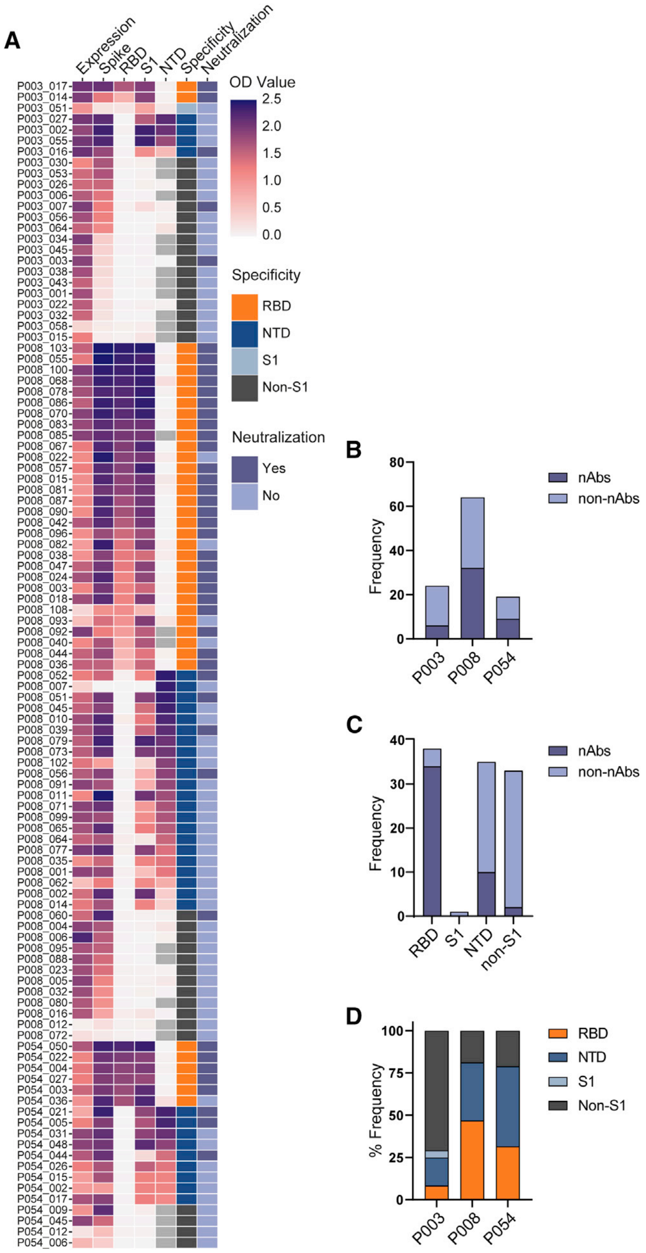

Figure 2. SARS-CoV-2 Spike reactive mAbs bind RBD, NTD, S1 and non-S1 epitopes

(A) Heatmap showing IgG expression level and binding to SARS-CoV-2 Spike, S1, NTD, and RBD. The figure reports OD values from a single experiment (range 0–2.5) for undiluted supernatant from small scale transfection of 107 cloned mAbs. Grey squares indicate samples that were not measured. Antigen binding was considered positive when OD at 405 nm was >0.3 after background was subtracted. SARS-CoV-2 Spike domain specificity for each antibody is indicated. Neutralization activity was measured against pseudotyped virus using either small-scale purified IgG or concentrated supernatant. Antibodies were considered neutralizing if at least 50% neutralization was reached at the highest concentration (5 μg/mL for purified mAb) or concentrated supernatant (~30 times).

(B) Bar graph showing frequency of nAbs and non-nAbs isolated from donors P003, P008, and P054.

(C) Bar graph showing frequency of nAbs and non-nAbs targeting specific Spike sub-domains.

(D) Bar graph showing the % of mAbs isolated from each donor targeting specific Spike sub-domains. See also Table S1.

CellPress
OPEN ACCESS

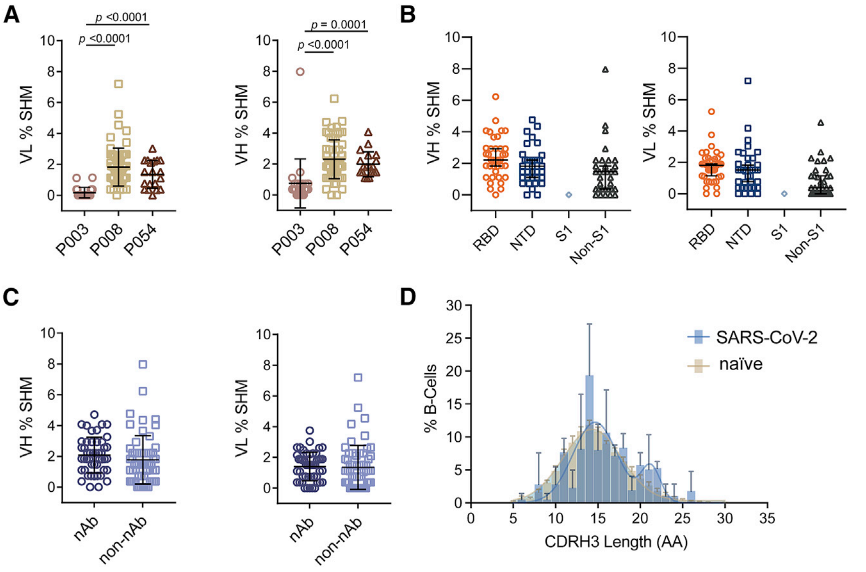

Figure 3. Sequence analysis of SARS-CoV-2 Spike specific mAbs shows diverse gene usage and low percentage somatic hypermutation

(A) Percentage SHM in the VH and VL genes of Spike-reactive mAbs for donors P003, P008, and P054. Differences between groups were determined using Kruskal-Wallis multiple comparison test and p values <0.05 are shown. Black lines represent the mean SHM and error bars represent the standard deviation.

(B) Percentage SHM for mAbs targeting RBD, NTD, S1, or non-S1 epitopes (Kruskal-Wallis multiple comparison test). Black lines represent the mean SHM and error bars represent the standard deviation.

(C) Percentage of VH and VL SHM for nAbs and non-nAbs (Mann-Whitney 2-sided U-test). Black lines represent the mean SHM and error bars represent the standard deviation.

(D) Distribution of CDRH3 lengths for SARS-CoV-2 specific mAbs and representative naive B cell repertoire (Briney et al., 2019). Error bars represent the standard deviation between donors used in the analysis (n = 3 for SARS-CoV-2 and n = 10 for naive repertoire). A bimodal distribution of CDRH3 length is observed for SARS-CoV-2 Spike reactive mAbs. Also see Figure S2.

SARS-CoV-2 mAbs have diverse gene usage

All Spike-reactive mAbs were sequenced, and their heavy and light germline gene usage, percentage somatic hypermutation (SHM), and CDRH3 length were determined using the ImmunoGenetics (IMGT) database (Brochet et al., 2008). A diverse range of heavy and light chain germline genes were utilized by mAbs isolated from the three donors (Figure S2B). Although an enrichment for certain germline genes was observed (Figures S2B and S2C), there was only one example of clonal expansion arising from donor P008 (Figure S2D). A comparison of VH gene usage compared to that of naive B cell repertoires (Briney et al., 2019) showed an enrichment in VH3-30 and, to a lesser extent, VH3-9 usage, and it showed a de-enrichment in VH3-23 and, to a lesser extent, VH3-7 and VH4-59 (Figure S2C). Despite the enrichment in VH3-30 gene usage, ten different light chains gene pairings were observed, including both kappa and lambda genes (Figure S2E). mAbs encoded by the VH3-66 and 3–53 germlines were frequently observed for RBD-specific mAbs as previously described (Figure S2A) (Barnes et al., 2020; Kim et al., 2021; Robbiani et al., 2020; Yuan et al., 2020a).

Overall, a low percentage SHM was observed in VH and VL genes (mean of 1.9% and 1.4%, respectively), which is expected following an acute viral infection. There was statistically higher SHM in VH of mAbs from P008 (2.3%) and P054 (2.0%), which used PBMCs isolated at days 61 and 48 POS respectively, compared to P003 (0.8%) which used PBMC isolated at day 20 POS (Figure 3A). There was no difference in the percentage SHM in the heavy or light chains between RBD-specific, NTD-specific and non-S1 mAbs (Figure 3B) or between neutralizing and non-neutralizing mAbs (Figure 3C). Comparison of the CDRH3 length distribution with representative naive repertoires showed an enrichment in CDRH3 of lengths 21 and 22 in the SARS-CoV-2-specific mAbs (Figure 3D). Overall, and similar to previously reported (Barnes et al., 2020; Brouwer et al., 2020; Jiang et al., 2020; Liu et al., 2020b; Pinto et al., 2020; Robbiani et al., 2020; Rogers et al., 2020; Tortorici et al., 2020; Wu et al., 2020b), the repertoire of SARS-CoV-2 specific mAbs was very diverse, did not differ greatly from that observed in representative naive repertoires, and showed very little SHM.

mAbs potently neutralize SARS-CoV-2

Representative neutralizing (n = 37) and non-neutralizing (n = 12) mAbs binding RBD, NTD, and non-S1 epitopes were selected for large-scale expression/purification and further characterization. The neutralization potency was measured against both pseudoviral particles and infectious virus (PHE strain using Vero-E6 as target cells). IC_{50} values ranged from 1.2–660 ng/mL against the pseudovirus particles and 2.3–488 ng/mL against infectious virus (Table S1).

RBD-specific mAb P008_108 is among the most potent anti-SARS-CoV-2 mAbs described so far with an IC_{50} of 2.3 ng/mL against infectious virus (Andreano et al., 2021). Although IC_{50} values measured against pseudovirus correlated well with those measured against infectious virus ($r = 0.7694$, $p < 0.0001$, Figure S3A), IC_{50} values were typically 5–10 fold less potent against infectious virus as seen previously with patient sera (Seow et al., 2020). Some S1/non-RBD nAbs that had shown weak neutralization (>10 μg/mL) against pseudovirus were only able to neutralize infectious virus at very high concentrations (>50 μg/mL) or undetectable neutralization (Figures 4B and 4C). In contrast, NTD-specific mAbs P008_056, P008_007 and P003_027 had ~10-fold higher potency against infectious virus (Table S1 and Figures S3C and S3D). In particular, P008_056 neutralized infectious virus with an IC_{50} of 14 ng/mL making this one of the most potent NTD nAbs reported thus far (McCallum et al., 2021). nAbs specific for RBD generally displayed more potent neutralization compared to those binding non-RBD epitopes (Figures 4B and 4C). Low neutralization plateaus and shallow neutralization curves were observed for some mAbs (Liu et al., 2020b) (Figures S3B and S3C) suggesting incomplete neutralization. These mAbs were typically less potent and had higher IC_{50} values >1,000 ng/mL. Seven of the non-S1 binding mAbs bound S2 in ELISA (Table

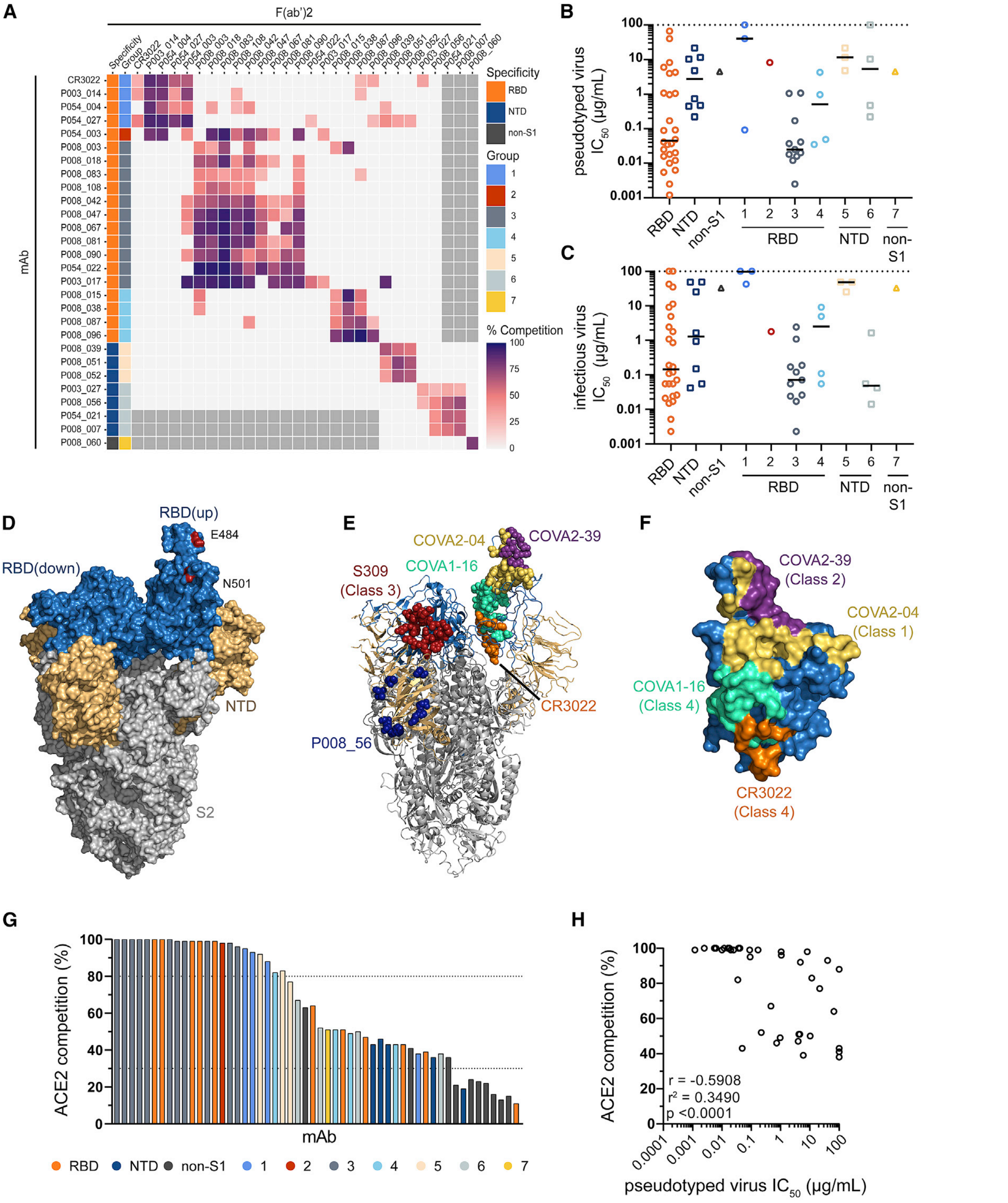

Figure 4. SARS-CoV-2 specific mAbs potently neutralize pseudovirus and infectious virus and form seven competition groups

(A–C) Inhibition of IgG binding to SARS-CoV-2 Spike by F(ab)$_2$' fragments was measured. The percentage competition was calculated using the reduction in IgG binding in the presence of F(ab')$_2$ (at 100-molar excess of the IC$_{80}$) as a percentage of the maximum IgG binding in the absence of F(ab')$_2$. Competition groups were determined using Ward2 clustering and clusters were then arranged according to binding epitopes. Experiments were performed in duplicate. Competition <25% is white. Grey boxes indicate competition not tested. Also see Figure S3G for competition with previously published mAbs (Brouwer et al., 2020). Neutralization potency (IC$_{50}$) of mAbs targeting either RBD, NTD or non-S1 and/or in competition Groups 1–7 against (B) SARS-CoV-2 pseudovirus and (C) infectious virus. Competition groups are color coded according to the key. The black lines represent the median IC$_{50}$ for each group. IC$_{50}$ values are the average of three independent experiments performed in duplicate.

(D–F) Mapping of previously determined neutralizing epitopes on RBD of SARS-CoV-2 Spike (PBD: 6XM0) (Zhou et al., 2020).

(legend continued on next page)

CellPress
OPEN ACCESS

S1), but none showed neutralizing activity. Overall, highly potent nAbs targeting RBD and NTD were identified.

nAbs form seven binding competition groups

To gain further insight into epitopes targeted by the isolated mAbs, we performed competition Spike ELISAs between 27 IgG and F(ab)$_2$' fragments (generated through IdeS digestion of purified IgG) (Figure 4A). In addition, to match competition groups to already identified neutralizing epitopes on Spike, we performed competition ELISAs with several SARS-CoV-2 specific IgG described by Brouwer et al. (Figure S3G) (Brouwer et al., 2020). Seven distinct competition groups were observed. nAbs binding RBD could be separated into four competition groups. Group 1 nAbs competed with a previously described SARS-CoV nAb CR3022 (ter Meulen et al., 2006) and COVA1-16, which both bind an RBD site distal to the ACE2 receptor binding site (Liu et al., 2020a; Yuan et al., 2020b; Yuan et al., 2020c) suggesting Group 1 nAbs can be classified as RBD Class 4 (Figures 4D–4F) (Barnes et al., 2020). Group 1 nAbs displayed limited neutralization potency, particularly against infectious virus (Table S1). Group 3 nAbs formed the largest and most potent competition group (Figures 4D–4F) containing 57.8% (11/19) of RBD nAbs tested in the competition ELISA, including the most potent mAb P008_108 (IC$_{50}$ 2.3 ng/mL against infectious virus). mAbs in this group were dominated (7/11) by the VH3-53 and VH3-66 germlines (Table S1 and Figure S2A). A similar VH3-53/VH3-66 gene enrichment has been reported for mAbs that directly bind the ACE2 receptor binding motif (RBM) on RBD (Barnes et al., 2020; Robbiani et al., 2020; Yuan et al., 2020a). Group 3 mAbs also competed with COVA2-04 (RBD Class 1 [Barnes et al., 2020]), and some competed to a lesser extent with COVA2-39 (RBD Class 2). Group 2 contained a single nAb with a CDRH3 of 22 amino acids that competed with antibodies in both Groups 1 and 3, suggesting an epitope overlapping competition Groups 1 and 3. This nAb also competed with COVA1-16 and COVA2-04. Group 4 contained four RBD reactive mAbs that formed a distinct competition group indicating a further distal RBD neutralizing epitope. Group 4 mAbs also competed with COVA2-02 (RBD Class 3 (Barnes et al., 2020)) which is thought to overlap with the S309 neutralizing epitope (Figures 4D–4F) (Pinto et al., 2020). Some RBD-specific nAbs did not compete with the COVA RBD-specific mAbs suggesting differences in epitope footprint and/or angle of antibody binding.

Non-RBD binding nAbs formed three competition groups. Group 5 contained three nAbs which bound Spike, S1 and NTD and had limited neutralization potency (IC$_{50}$ 4.8–21.7 μg/mL and 25.3–48.8 μg/mL against pseudovirus and infectious virus, respectively). Group 6 contained four nAbs, which were all more potent against infectious virus compared to pseudovirus (Figures 4B and 4C) and included P008_056, which neutralized infectious virus with an IC$_{50}$ (14 ng/mL), in line with the most potent RBD binding nAbs. Structural analysis revealed that P008_056 binds NTD adjacent to the β sandwich fold (Figure 4E) (Rosa et al., 2021). The NTD nAbs showed minimal or no competition with NTD nAbs COVA1-22 or COVA2-17 (Figure S3G). Group 7 contained only one nAb, P008_060, which bound to Spike and not individual S1 or S2 domains, suggesting that it may target a quaternary epitope spanning multiple domains, similar to 2-43 (Liu et al., 2020b). P008_060 did not compete with non-RBD mAbs COVA1-26, COVA1-12, or COVA3-08 (Figure S3G) (Brouwer et al., 2020).

Overall, seven competition groups were identified. RBD nAbs bound epitopes overlapping with previously characterized RBD antibody classes. However, further structural characterization will be needed to fully define the neutralizing epitopes of NTD-specific and non-S1-specific nAbs.

mAbs inhibit Spike-ACE2 interaction to differing extents

To explore the potential mechanism by which nAbs prevent infection of target cells, we measured the ability of nAbs to prevent the interaction of Spike with the ACE2 receptor on HeLa cells by flow cytometry (Figure 4G). Group 3 nAbs showed >99% inhibition of Spike binding to HeLa-ACE2 cells, suggesting that these nAbs directly target the ACE2 binding site on RBD. Overall, nAbs displaying the highest competition with ACE2 binding typically had the highest neutralization potency (Figure 4H). Similar to CR3022, Group 1 nAbs showed less complete competition (88.2%–95.1%), and Group 4 mAbs show only partial competition (43.1%–82.2%), suggesting that these nAbs can sterically inhibit the interaction of Spike with ACE2 without directly binding to the RBM or cause conformational changes to Spike that limit ACE2 binding. NTD-binding, and Spike-specific nAbs (Groups 5, 6, and 7) also showed some partial competition (38.4%–91.8%). Although these nAbs do not compete with RBD nAbs, it is possible that binding to Spike causes conformational changes that prevent subsequent ACE2 binding or lock RBD in the "down" conformation, which occludes access to the ACE2 binding site (Liu et al., 2020b). As might be expected, S2-reactive mAbs and S1-reactive non-neutralizing mAbs showed negligible competition with ACE2 binding. Overall, these results suggest that some antibodies described here neutralize SARS-CoV-2 through mechanisms beyond direct receptor

(D) Surface rendered representation of SARS-CoV-2 Spike (side view) showing the RBD (blue), NTD (brown) and S2 (gray) domains. One RBD monomer is in the "up" conformation. Positions of Spike mutations relevant to neutralization escape (N501Y and E484K) are indicated in red.

(E) Cartoon representation of Spike showing antibody binding footprint for nAbs used in competition ELISAs as colored spheres. Epitopes were previously determined using crystal structures or cryo-electron microscopy of RBD or Spike-Fab complexes; COVA2-04 (yellow, RBD Class 1, [PBD: 7JMO] [Wu et al., 2020a]), COVA2-39 (purple, RBD Class 2 [PBD: 7JMP] [Wu et al., 2020a]), S309 (red, RBD Class 3 [PBD: 6WPS] [Pinto et al., 2020]), COVA1-16, and CR3022 (pale green [PBD: 7JMW] [Liu et al., 2020a] and orange [PBD: 6W41] [Yuan et al., 2020c], respectively, RBD Class 4), and P008_056 (dark blue, NTD [Rosa et al., 2021]).

(F) Surface representation of zoomed in RBD in "up" conformation showing footprint of RBD nAbs. Structures were generated in Pymol using the referenced PBDs.

(G) Ability of nAbs and non-nAbs to inhibit the interaction between cell surface ACE2 and soluble SARS-CoV-2 Spike. mAbs (at 600 nM) were pre-incubated with fluorescently labeled Spike before addition to HeLa-ACE2 cells. The percentage reduction in mean fluorescence intensity is reported. Experiments were performed in duplicate. Bars are color coded based on their competition group or binding specificity if competition group was not determined.

(H) Correlation between IC$_{50}$ against pseudovirus and % ACE2 competition. (Spearman correlation, r. A linear regression was used to calculate the goodness of fit, r^2). Also see Figures S3A–S3D and Table S1.

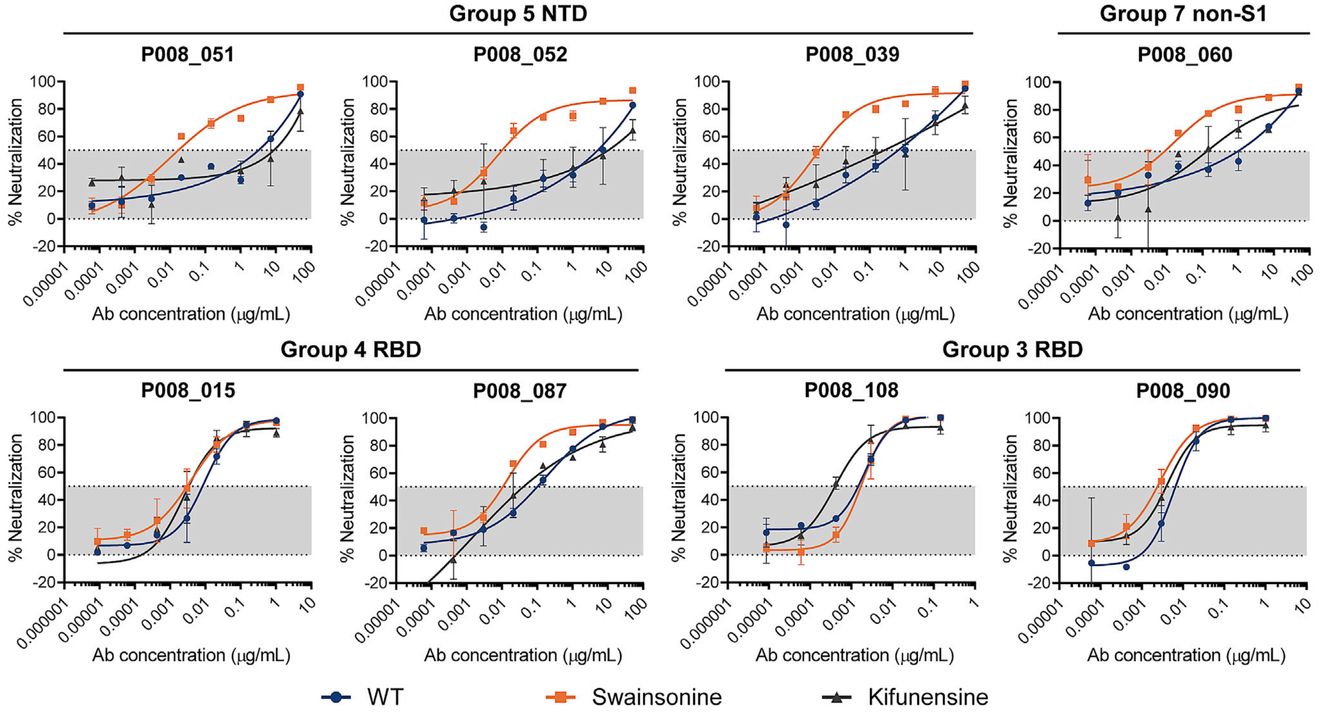

Figure 5. Neutralization of Group 5 nAbs enhanced by changes in Spike glycosylation

SARS-CoV-2 pseudovirus was expressed in the presence of glycosidase inhibitors kifunensine or swainsonine. Neutralization potency of RBD and NTD nAbs against Spike-modified pseudoviruses was measured. Group 5 NTD nAbs (P008_051, P008_052, and P008_039) and Group 7 non-S1 nAb (P008_060) showed an enhanced neutralization potency and more typical shaped neutralization curve compared to Spike with wild-type glycans. In contrast, RBD nAbs (P008_015, P008_087, P008_108, and P008_090) had unchanged neutralization. Neutralization assays were performed three times in duplicate and a representative experiment is shown.

binding inhibition, such as inhibiting membrane fusion (McCallum et al., 2021) or S1 shedding (Piccoli et al., 2020), which need be investigated further.

Glycan heterogeneity influences neutralization potency

As mentioned above, some nAbs displayed shallow neutralization curves that plateau below 100% neutralization against pseudovirus, and this was more typical for mAbs specific for NTD. Similar unusual neutralization profiles have been observed for some HIV-1 bnAbs, in particular, those that accommodate and/or bind *N*-linked glycans on the HIV-1 Env surface, and is thought to arise due to heterogeneity in glycosylation (Doores and Burton, 2010). This phenotype could be rescued for some HIV-1 bnAbs by altering the composition of Env glycans by expressing virus in the presence of glycosidase inhibitors such as kifunensine (that inhibits the ER-mannosidase I enzyme leading to $Man_9GlcNAc_2$ glycans) and swainsonine (that inhibits the Golgi-α-mannosidase II enzyme, leading to truncated complex-type glycans in addition to the naturally occurring high-mannose glycans present).

As NTD is heavily glycosylated (Watanabe et al., 2020), we next investigated whether changes in the glycan structures on Spike, through preparation of pseudovirus in the presence of glycosidase inhibitors kifunensine or swainsonine, could affect neutralization activity. RBD mAbs, P008_015, P008_087, P008_090, and P008_108, were not impacted by alterations in Spike glycan processing (Figure 5). However, NTD-specific Group 5 mAbs, P008_039, P008_051, and P008_052, and non-S1 Group 7 mAb, P008_060, showed enhanced neutralization against pseudovirus prepared in the presence of swainsonine where glycan structures will be smaller in size. No change in neutralization was observed against pseudovirus prepared with kifunensine, although lower infectivity was noted as previously reported (Yang et al., 2020). These data suggest that glycan structures can affect nAb epitope recognition either through modulating the conformation of Spike or altering the accessibility of nAb epitopes.

Some nAbs display cross-reactivity with SARS-CoV

SARS-CoV Spike shares 73% sequence homology with Spike of SARS-CoV-2 and 73% with RBD (Walls et al., 2020). To determine whether isolated mAbs targeted epitopes shared between SARS-CoV-2 and SARS-CoV, cross-neutralization against SARS-CoV was measured using the HIV-1 pseudovirus system expressing full-length SARS-CoV Spike. Although neutralization was detected for several mAbs, this was generally at a much reduced potency (3- to 65-fold) compared to SARS-CoV-2 neutralization (Table S1). nAb CR3022, isolated following SARS-CoV infection, has previously been reported to bind an epitope conserved between SARS-CoV and SARS-CoV-2 (Yuan et al., 2020c). However, cross-neutralization was observed for only one nAb in the CR3022 competition group (Group 1) and single nAbs from Groups 4 (RBD), 5 (NTD) and 7 (non-S1), suggesting nAbs within each competition group differ in their molecular contacts. In contrast, the sole mAb in Group

7, which reacts only with Spike trimer and not individual subunits, showed a 7-fold more potent neutralization against SARS-CoV compared to SARS-CoV-2. Binding of nAbs to SARS-CoV Spike expressed on the surface of HEK293T cells was detected for mAbs showing SARS-CoV neutralizing activity but not by SARS-CoV non-nAbs in the same competition groups (Figure S3E). However, S2-binding non-nAbs, although unable to neutralize SARS-CoV (Table S1), bound to cell surface expressed SARS-CoV Spike (Figure S3F), indicating the presence of a conserved, non-neutralizing S2 epitope. Whether the S2 mAbs can bind SARS-CoV-2 infected cells and recruit ADCC *in vivo* is not known (Li et al., 2020b). Overall, conserved neutralizing epitopes shared between SARS-CoV-2 and SARS-CoV are present on both RBD and NTD.

nAbs from distinct competition groups are differentially impacted by emerging Spike variants

The SARS-CoV-2 D614G Spike variant supplanted the ancestral virus in most areas worldwide early in the pandemic, and although the mutation has been reported to be more infectious through stabilization of RBD in the "up" conformation, it has not been associated with neutralization escape (Li et al., 2020a; Weissman et al., 2020; Yurkovetskiy et al., 2020). More recently the B.1.1.7 variant of concern (VOC 202012/01) first reported in the UK, which contains an additional eight Spike mutations in NTD, RBD, and S2 (ΔH69/V70, ΔY144, N501Y, A570D, P681H, T716I, S982A, D1118H) (Rambaut et al., 2020), has been associated with more efficient transmission within the UK and is now the dominant variant in London and the South East of England (Rambaut et al., 2020). It is not known whether these mutations have arisen stochastically, have been selected purely on the basis of increased transmission, or whether the emergence of B.1.1.7 was in part driven by the pressure of nAbs in longer term infections in immunocompromised patients undergoing passive immunotherapy (Kemp et al., 2021; McCarthy et al., 2021). Nor is it clear if it will lead to escape from nAbs generated in response to SARS-CoV-2 infection in wave 1 and/or generated through vaccination. Initial reports have suggested that the B.1.1.7 variant is sensitive to polyclonal sera from individuals infected with early circulating SARS-CoV-2 variants (Collier et al., 2021; Edara et al., 2021; Rees-Spear et al., 2021; Shen et al., 2021; Supasa et al., 2021; Wang et al., 2021; Zhou et al., 2021).

We measured neutralization potency of nAbs from the seven competition groups, as well as patient plasma from P008 and P054, against HIV-1 viral particles pseudotyped with SARS-CoV-2 Spike bearing mutations D614G, N501Y (part of the ACE2 receptor binding site and associated with increased transmission and enhanced ACE2 binding affinities [Starr et al., 2021]), D614G+ΔH69/V70, D614G+ΔY144, and the B.1.1.7 variant containing all eight Spike mutations. Similar to previous data (Yurkovetskiy et al., 2020), the D614G mutation alone had a limited effect on neutralization by the majority of RBD nAbs. Conversely, NTD nAbs within competition group 5 showed a 19–450-fold decrease in neutralization potency (Figure 6A and Figure S4A). Furthermore, Group 4 RBD nAbs showed a 3–22-fold decrease in potency, and Group 1 RBD nAb, P054_027, showed a 25-fold reduction. Despite the location of the N501Y mutation in RBD (Figure 4D), the vast majority of RBD-specific nAbs were not affected by this mutation (Figure 6A and Figure S4A). By contrast, group 3 nAbs P003_017 and P008_003 showed a 25- and 9-fold decrease respectively, and group 1 nAbs P054_004 and P054_027 showed a 14- and 8-fold decrease respectively in neutralization potency. Minimal changes in neutralization potency were observed for Group 5 and 6 NTD mAbs against the N501Y mutant.

The ΔH69/V70 and ΔY144, situated within the N1 loop of NTD, have been associated with viral evolution in immunocompromised SARS-CoV-2-infected individuals (Kemp et al., 2021; McCarthy et al., 2021). D614G+ΔH69/V70 had a limited effect on neutralization of RBD-specific or NTD-specific antibodies, with only P003_027 showing a 16-fold reduction when compared to the D614G variant (Figure 6A and Figure S4B). In contrast, D614G+ΔY144 completely abolished neutralization activity by Group 6 NTD nAbs and showed a 7–15-fold reduction for Group 5 NTD nAbs (Figure 6B). Some RBD nAbs showed up to a 7-fold reduction in neutralization potency.

Similar to the D614G+ΔY144 mutant, Group 6 NTD nAbs showed no neutralizing activity up to 100 μg/mL against the B.1.1.7 variant (Figure 6B and Figure S4B) and only weak neutralization by Group 5 NTD nAbs was measured (IC_{50} 30-100 μg/mL). The most potent RBD nAbs in Groups 3 and 4 showed no reduction in neutralization activity against the B.1.1.7 variant. In contrast, P008_081 and P054_022 (Group 3) showed a 6- and 18- fold reduction against B.1.1.7 while maintaining IC_{50} values of 0.34 and 2.8 μg/mL range (Figure S4A), respectively. RBD mAbs P003_017 (Group 3) and P054_027 (Group 1) showed the greatest reduction in neutralization with only very weak neutralization detected at 50 μg/mL.

An additional E484K mutation, which is of particular concern for neutralization resistance in the B.1.351 variant prevalent in South Africa (Wang et al., 2021; Wibmer et al., 2021), has been observed in combination with B.1.1.7 mutations and identified as a variant of concern in the UK (VOC 202102/02) (Collier et al., 2021; Wise, 2021). To determine whether this additional mutation would lead to neutralization resistance by RBD nAbs, we measured neutralization sensitivity against the B.1.1.7+E484K Spike variant. Neutralization of NTD-specific nAbs was unchanged in relation to the B.1.1.7 variant. Group 3 RBD-specific nAbs, which directly compete with ACE2 binding, and are therefore likely to overlap the E484K mutation, showed differing sensitivities to B.1.1.7+E484K. P054_022, P003_017, and P008_003 showed no neutralization activity whereas the remaining Group 3 nAbs showed a 4–9-fold reduction in IC_{50} compared to D614G with only a maximum neutralization of ~80% reached (Figure 6B and Figure S4B). Group 4 nAb, P008_015 showed >500-fold reduction in neutralization, whereas other Group 4 nAbs showed a more modest 3–9 fold reduction compared to D614G (Figure 6B). Some RBD nAbs still potently neutralized the B.1.1.7+E484K variant with IC_{50} values as low as 0.008 μg/mL.

Despite the reduction in neutralization potency or loss of neutralization activity for specific mAbs, neutralization potency of sera from P008 (61 days POS) showed an 8-fold reduction in potency against the B.1.1.7 variant (Figure 6C), suggesting that the polyclonal nature of the antibody response overcomes the Spike mutations in the B.1.1.7 variant. However, sera from P054 (48 days POS), which was unaffected by B.1.1.7 mutations, showed an 8-fold reduction in potency against B.1.1.7+E484K.

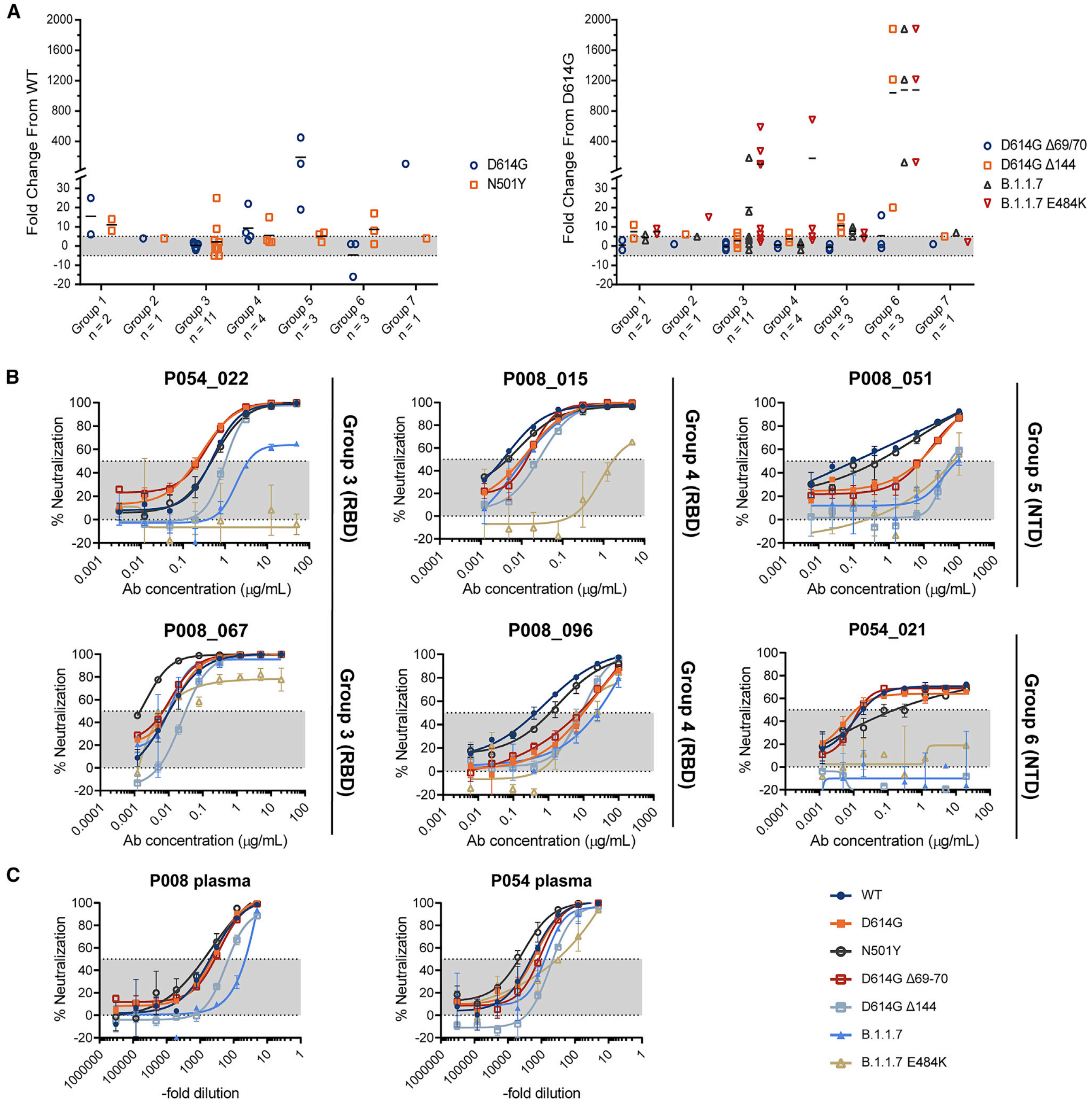

Figure 6. Some nAbs show reduced neutralization potency against B.1.1.7 and related Spike variants

Neutralization by mAbs and plasma were tested against pseudoviruses expressing variant Spikes.

(A) Fold change in neutralization potency for D614G and N501Y mutation compared to wild-type Spike, and D614G+ΔH69/V70, D614G+ΔH69/V70, B.1.1.7, and B.1.1.7+E484K variants compared to D614G Spike. Black lines represent the mean fold change for each competition Group. IC_{50} values were calculated from two independent experiments and used to caluclate fold change.

(B) Example neutralization curves for Group 3, 4, 5, and 6 nAbs against Spike variants.

(C) Neutralization activity in P008 and P054 plasma against Spike variants at B cell sorting time point. Also see Figures S4 and S5.

Overall, NTD-specific nAbs showed the greatest reduction in neutralization potency against the B.1.1.7 Spike variant, facilitated by the ΔY144 mutation. Although some RBD-specific nAbs also showed a reduction in potency, potent neutralization was still observed, even against the B.1.1.7+E484K variant of concern.

DISCUSSION

Here, we isolated SARS-CoV-2 Spike reactive neutralizing and non-neutralizing antibodies from three convalescent donors who experienced a range of COVID-19 illness severities. As

CellPress
OPEN ACCESS

observed previously (Barnes et al., 2020; Brouwer et al., 2020; Jiang et al., 2020; Liu et al., 2020b; Pinto et al., 2020; Robbiani et al., 2020; Rogers et al., 2020; Tortorici et al., 2020; Wu et al., 2020b), the antibody response to SARS-CoV-2 is very diverse, is not restricted to specific germlines, and does not require extensive SHM for neutralization as seen for HIV-1 nAbs (Doores et al., 2015; Scheid et al., 2009). Antibodies against RBD, NTD, and non-S1 epitopes were isolated from all three donors. The most potent nAb, P008_108 (IC_{50} 2.3 ng/mL against infectious virus), was isolated from an asymptomatic donor with very low serum neutralizing activity. Isolation of similar potent mAbs from donors with low serum neutralizing activity has also been reported by Robbiani et al. (Robbiani et al., 2020) and suggests that the lower neutralization potency of sera from individuals experiencing mild or no COVID-19 symptoms is due to a low quantity of plasma neutralizing antibody rather than sub-optimal potency of individual antibodies. It also suggests that memory responses are not proportional to the antibodies arising in serum after the immediate plasmablast burst. Upon re-exposure, all individuals would be expected to produce highly potent nAbs.

The use of Spike for antigen-specific B cell sorting allowed us to isolate mAbs targeting epitopes beyond RBD and to study their relevance in viral evolution and antigenic changes in Spike. Roughly one third of isolated mAbs were specific for NTD, but only 28.5% of these showed neutralizing activity. Neutralizing NTD mAbs formed two distinct competition groups. The most potent competition group (Group 6) contained nAbs that neutralized infectious virus more potently than pseudovirus. Assuming the structural conformations and dynamics of the Spike on pseudovirus and infectious virus are identical, the discordant neutralization differences may relate to differences in Spike density, Spike glycan heterogeneity or expression level of the ACE2 receptor on the target cell lines used in the two assays. nAb P008_056 neutralized infectious virus with an IC_{50} of 14 ng/mL, making this one of the most potent NTD nAbs reported (Rogers et al., 2020) and in line with the most potent RBD nAb isolated here. Structural analysis of the interaction of P008_056 Fab with Spike revealed that the nAb binds the viral glycoprotein at the distal face of the NTD, including substantial conformation changes in this domain (Rosa et al., 2021). The less potent NTD competition group (Group 5) showed atypical neutralization curves. For these nAbs, neutralization activity could be enhanced by reducing the size and/or composition of *N*-glycans on Spike through preparation of pseudoviral particles in the presence of swainsonine. As NTD is heavily glycosylated, a reduction in the glycan size will likely increase the accessibility of protein epitopes on NTD (Watanabe et al., 2020) and thus enhance binding efficiency and neutralization potency.

RBD-specific nAbs isolated here targeted epitopes similar to those reported previously (Barnes et al., 2020; Brouwer et al., 2020; Piccoli et al., 2020; Seydoux et al., 2020; Yuan et al., 2020b; Zost et al., 2020) and included nAbs that directly block ACE2 binding through binding RBM and nAbs targeting epitopes distal to the RBM. We also isolated a high proportion of Spike reactive mAbs, which showed no neutralizing activity demonstrating the presence of immunodominant, non-neutralizing epitopes on RBD, NTD, and S2. 31.8% (34/107) of mAbs did not bind S1, suggesting S2 or quaternary epitopes, but only three showed neutralizing activity. However, non-neutralizing S2 reactive mAbs were able to cross-react with SARS-CoV Spike expressed on the surface of cells. As the non-neutralizing mAbs are able to bind cell-surface expressed Spike, it will be important to investigate whether they can facilitate Fc effector functions such as ADCC and play a role in virus clearance (Bournazos et al., 2020; Li et al., 2020b).

SARS-CoV-2 variants are rapidly emerging across the globe, and it is important to determine whether antibodies generated during wave 1 infections or following vaccination will provide protection against these emerging variants of concern (Rambaut et al., 2020). Despite the dominant nAb response being against RBD, we show that the B.1.1.7 variant is still potently neutralized by the majority of RBD-specific nAbs but is resistant to NTD-specific nAbs, findings supported by other recent publications and preprints (Chen et al, 2021; Wang et al., 2021). The ΔY144 deletion facilitates neutralization escape of Group 6 NTD nAbs in the B.1.1.7 variant. Indeed, structural analysis of P008_056 in complex with SARS-CoV-2 Spike shows that Y144 sits within a loop that must undergo conformational rearrangement to allow access to the P008_056 epitope on NTD (Rosa et al., 2021). It is not possible to conclude from our data whether nAbs against NTD are selecting for Spike variants encoding NTD deletions, such as B.1.1.7, and/or whether NTD mutations alter Spike functionality to favor increased transmissibility. More specifically, deletions in NTD have been associated with neutralization escape from mAbs (Andreano et al., 2020; Kemp et al., 2021; McCarthy et al., 2021; Wibmer et al., 2021). The ΔY144 deletion, which has a prevalence of 1.7% in 37 countries (McCallum et al., 2021), has been shown to abrogate binding to other NTD mAbs including S2M28, S2X28, S2X333, and 4A8 (Chi et al., 2020; McCallum et al., 2021). NTD deletions, including ΔH69/V70 (Kemp et al., 2021) and Δ141–144 (Avanzato et al., 2020; Choi et al., 2020; McCarthy et al., 2021), have been observed in immunocompromised individuals who remain infected for extended periods. Lastly, deletion of NTD residues 242–244 from the B.1.351 variant (501Y.V2 prevalent in South Africa) has been shown to reduce binding by NTD-specific mAbs 4A8 (Wibmer et al., 2021) and 4-8 (Wang et al., 2021). Thus, more research is needed to establish the driver for the observed accumulation of genetic changes in the Spike of circulating SARS-CoV-2 strains.

Despite the loss in neutralization of NTD-specific nAbs against B.1.1.7, neutralization by RBD-specific nAbs either remained unchanged or, when a reduction was observed, neutralization in the 0.001–5 μg/mL range was still measured for the majority of antibodies. The reduction in RBD-specific mAbs neutralization against B.1.1.7 was of lower magnitude than that reported for mAbs against the B.1.351 variant (Collier et al., 2021; Wang et al., 2021; Wibmer et al., 2021; Zhou et al., 2021). The B.1.351 variant also encodes RBD mutations K417N and E484K, which have been associated with viral escape from RBD-targeting antibodies (Starr et al., 2021; Weisblum et al., 2020). As RBD is the predominant target for nAbs following infection (Greaney et al., 2021; Piccoli et al., 2020), this would suggest RBD-specific nAbs had a limited contribution to any immune escape contributing to the selection of the B.1.1.7 variant. Importantly, although there was an 8-fold decrease in P008 plasma neutralization against the B.1.1.7 variant, neutralization could still be detected, and neutralization by P054 plasma was

unchanged. This suggests that although complete loss of neutralization is observed for mAbs targeting specific epitopes, further mutations would be needed for complete neutralization escape from the polyclonal antibody response generated from SARS-CoV-2 infection in these individuals, which is supported by larger scale studies using convalescent plasma (Rees-Spear et al., 2021; Shen et al., 2021; Supasa et al., 2021; Wang et al., 2021; Zhou et al., 2021). Indeed, introduction of the E484K mutation to the B.1.1.7 variant further reduced neutralization potency of some RBD nAbs and plasma but did not lead to widespread resistance (Collier et al., 2021).

In conclusion, we identified potent nAbs targeting both RBD and NTD neutralizing epitopes. We show that the B.1.1.7 variant is resistant to neutralization by the NTD nAbs demonstrating the importance of considering both dominant and sub-dominant neutralizing epitopes on Spike when studying viral evolution and antigenic drift.

Limitations of study

Here we isolated 107 mAbs from three SARS-CoV-2 convalescent individuals. This small number may not be representative of the SARS-CoV-2 specific repertoire, and rare nAbs may have been missed. Although epitope information was obtained through competition ELISAs, structural analysis is required to further characterize the neutralizing epitopes of nAbs. Finally, due to the ease of incorporating Spike variants, neutralization against B.1.1.7 and related mutants was measured with pseudovirus and not infectious virus.

STAR METHODS

Detailed methods are provided in the online version of this paper and include the following:

- KEY RESOURCES TABLE
- RESOURCE AVAILABILITY
  - Lead contact
  - Materials availability
  - Data and Code availability
- EXPERIMENTAL MODEL AND SUBJECT DETAILS
  - Human subjects
  - Bacterial strains and cell culture
  - Viruses
- METHOD DETAILS
- QUANTIFICATION AND STATISTICAL ANALYSIS

SUPPLEMENTAL INFORMATION

Supplemental information can be found online at https://doi.org/10.1016/j.immuni.2021.03.023.

ACKNOWLEDGMENTS

This work was funded by; King's Together Rapid COVID-19 Call award to M.H.M., K.J.D., and S.J.D.N., UK Medical Research Council (MRC) Discovery award MC/PC/15068 to S.J.D.N., K.J.D., and M.H.M., Fondation Dormeur, Vaduz for funding equipment to K.J.D., a Huo Family Foundation award to M.H.M., K.J.D., M.S.H., and S.J.D.N., MRC Programme Grant (MR/S023747/1 to M.H.M.), Wellcome Trust Multi-User Equipment Grant 208354/Z/17/Z to M.H.M., S.J.D.N., K.D., and A.C.H., Wellcome Trust Investigator Award 106223/Z/14/Z to M.H.M., NIAID Awards U54 AI150472 and AI076119 to M.H.M., The UK CIC (Covid-Immunology-Consortium) to A.C.H., the Rosetrees Trust to A.C.H., and UCL Coronavirus Response Fund to L.E.M. (made possible through generous donations from UCL's supporters, alumni, and friends). M.S.H. is funded by the National Institute for Health Research Clinician Scientist Award (CS-2016-16-011). The views expressed in this publication are those of the author(s) and not necessarily those of the NHS, the National Institute for Health Research or the Department of Health and Social Care. This study is part of the EDCTP2 program supported by the European Union (grant number RIA2020EF-3008 COVAB). The views and opinions of authors expressed herein do not necessarily state or reflect those of EDCTP. C.G. and H.W. were supported by the MRC-KCL Doctoral Training Partnership in Biomedical Sciences (MR/N013700/1). S.A. was supported by an MRC-KCL Doctoral Training Partnership in Biomedical Sciences industrial Collaborative Award in Science & Engineering (iCASE) in partnership with Orchard Therapeutics (MR/R015643/1). Work in P.C. laboratory is funded by the Francis Crick Institute (FC001061), which receives its core funding from Cancer Research UK, the UK Medical Research Council, and the Wellcome Trust. T.A.B. is supported by the Medical Research Council (MR/S007555/1). The Wellcome Centre for Human Genetics is supported by Wellcome Centre grant 203141/Z/16/Z. L.E.M. was supported by a Medical Research Council Career Development Award MR/R008698/1. This work was supported by the Department of Health via a National Institute for Health Research comprehensive Biomedical Research Centre award to Guy's and St. Thomas' NHS Foundation Trust in partnership with King's College London and King's College Hospital NHS Foundation Trust.

Thank you to Philip Brouwer and Rogier Sanders (University of Amsterdam) for the Spike protein construct, and James Voss and Deli Huang for providing the HeLa-ACE2 cells.

AUTHOR CONTRIBUTIONS

Conceptualization, K.J.D., M.H.M., C.G., J.S.; Methodology, C.G., J.S., I.H., H.K., N.K., S.A., H.W., S.P., R.P.G, L.D., M.J.L., J.M.J.-G., L.M., W.M.N., H.M.E.D., Y.Z., T.A.B.; Formal analysis, K.J.D., C.G., J.S., I.H., H.K., W.N.G., T.A.B.; Resources, A.G.L., Y.W., M.J., M.S.-H., A.R., P.C., L.E.M., A.C.H., M.J.v.G.; Supervision, K.J.D., M.H.M., S.J.D., A.C.H., L.E.M., P.C.; Writing – original draft, K.J.D., C.G., J.S., I.H., H.K., M.H.M.; All authors reviewed and edited the manuscript.

DECLARATION OF INTERESTS

The authors declare no competing interests.

Received: February 3, 2021
Revised: March 5, 2021
Accepted: March 29, 2021
Published: April 1, 2021

REFERENCES

Andreano, E., Piccini, G., Licastro, D., Casalino, L., Johnson, N.V., Paciello, I., Monego, S.D., Pantano, E., Manganaro, N., Manenti, A., et al. (2020). SARS-CoV-2 escape *in vitro* from a highly neutralizing COVID-19 convalescent plasma. bioRxiv, 2020.12.28.424451.

Andreano, E., Nicastri, E., Paciello, I., Pileri, P., Manganaro, N., Piccini, G., Manenti, A., Pantano, E., Kabanova, A., Troisi, M., et al. (2021). Extremely potent human monoclonal antibodies from COVID-19 convalescent patients. Cell, S0092-8674(21)00224-5.

Aricescu, A.R., Lu, W., and Jones, E.Y. (2006). A time- and cost-efficient system for high-level protein production in mammalian cells. Acta Crystallogr. D Biol. Crystallogr. *62*, 1243–1250.

Avanzato, V.A., Matson, M.J., Seifert, S.N., Pryce, R., Williamson, B.N., Anzick, S.L., Barbian, K., Judson, S.D., Fischer, E.R., Martens, C., et al. (2020). Case Study: Prolonged Infectious SARS-CoV-2 Shedding from an Asymptomatic Immunocompromised Individual with Cancer. Cell *183*, 1901–1912.e1909.

Barnes, C.O., Jette, C.A., Abernathy, M.E., Dam, K.A., Esswein, S.R., Gristick, H.B., Malyutin, A.G., Sharaf, N.G., Huey-Tubman, K.E., Lee, Y.E., et al. (2020).

CellPress
OPEN ACCESS

SARS-CoV-2 neutralizing antibody structures inform therapeutic strategies. Nature *588*, 682–687.

Beaudoin-Bussières, G., Laumaea, A., Anand, S.P., Prévost, J., Gasser, R., Goyette, G., Medjahed, H., Perreault, J., Tremblay, T., Lewin, A., et al. (2020). Decline of Humoral Responses against SARS-CoV-2 Spike in Convalescent Individuals. MBio *11*, e02590-20.

Bournazos, S., Corti, D., Virgin, H.W., and Ravetch, J.V. (2020). Fc-optimized antibodies elicit CD8 immunity to viral respiratory infection. Nature *588*, 485–490.

Briney, B., Inderbitzin, A., Joyce, C., and Burton, D.R. (2019). Commonality despite exceptional diversity in the baseline human antibody repertoire. Nature *566*, 393–397.

Brochet, X., Lefranc, M.P., and Giudicelli, V. (2008). IMGT/V-QUEST: the highly customized and integrated system for IG and TR standardized V-J and V-D-J sequence analysis. Nucleic Acids Res. *36*, W503-8.

Brouwer, P.J.M., Caniels, T.G., van der Straten, K., Snitselaar, J.L., Aldon, Y., Bangaru, S., Torres, J.L., Okba, N.M.A., Claireaux, M., Kerster, G., et al. (2020). Potent neutralizing antibodies from COVID-19 patients define multiple targets of vulnerability. Science *369*, 643–650.

Carter, M.J., Fish, M., Jennings, A., Doores, K.J., Wellman, P., Seow, J., Acors, S., Graham, C., Timms, E., Kenny, J., et al. (2020). Peripheral immunophenotypes in children with multisystem inflammatory syndrome associated with SARS-CoV-2 infection. Nat. Med. *26*, 1701–1707.

Characterisation, W.H.O.W.G.C.; WHO Working Group on the Clinical Characterisation and Management of COVID-19 infection (2020). A minimal common outcome measure set for COVID-19 clinical research. Lancet Infect. Dis. *20*, e192–e197.

Chen, R.E., Zhang, X., Case, J.B., Winkler, E.S., Liu, Y., VanBlargan, L.A., Liu, J., Errico, J.M., Xie, X., Suryadevara, N., et al. (2021). Resistance of SARS-CoV-2 variants to neutralization by monoclonal and serum-derived polyclonal antibodies. Nat. Med.

Chi, X., Yan, R., Zhang, J., Zhang, G., Zhang, Y., Hao, M., Zhang, Z., Fan, P., Dong, Y., Yang, Y., et al. (2020). A neutralizing human antibody binds to the N-terminal domain of the Spike protein of SARS-CoV-2. Science *369*, 650–655.

Choi, B., Choudhary, M.C., Regan, J., Sparks, J.A., Padera, R.F., Qiu, X., Solomon, I.H., Kuo, H.H., Boucau, J., Bowman, K., et al. (2020). Persistence and Evolution of SARS-CoV-2 in an Immunocompromised Host. N. Engl. J. Med. *383*, 2291–2293.

Collier, D.A., De Marco, A., Ferreira, I.A.T.M., Meng, B., Datir, R., Walls, A.C., Kemp S, S.A., Bassi, J., Pinto, D., Fregni, C.S., et al.; CITIID-NIHR BioResource COVID-19 Collaboration; COVID-19 Genomics UK (COG-UK) consortium (2021). Sensitivity of SARS-CoV-2 B.1.1.7 to mRNA vaccine-elicited antibodies. Nature.

Crawford, K.H.D., Dingens, A.S., Eguia, R., Wolf, C.R., Wilcox, N., Logue, J.K., Shuey, K., Casto, A.M., Fiala, B., Wrenn, S., et al. (2020). Dynamics of neutralizing antibody titers in the months after SARS-CoV-2 infection. J. Infect. Dis. jiaa618.

Dan, J.M., Mateus, J., Kato, Y., Hastie, K.M., Yu, E.D., Faliti, C.E., Grifoni, A., Ramirez, S.I., Haupt, S., Frazier, A., et al. (2021). Immunological memory to SARS-CoV-2 assessed for up to 8 months after infection. Science *371*, eabf4063.

Dixon, E.V. (2014). Mechanisms of immunoglobulin deactivation by Streptococcus pyogenes, PhD Thesis.

Doores, K.J., and Burton, D.R. (2010). Variable loop glycan dependency of the broad and potent HIV-1-neutralizing antibodies PG9 and PG16. J. Virol. *84*, 10510–10521.

Doores, K.J., Kong, L., Krumm, S.A., Le, K.M., Sok, D., Laserson, U., Garces, F., Poignard, P., Wilson, I.A., and Burton, D.R. (2015). Two classes of broadly neutralizing antibodies within a single lineage directed to the high-mannose patch of HIV envelope. J. Virol. *89*, 1105–1118.

Edara, V.V., Floyd, K., Lai, L., Gardner, M., Hudson, W., Piantadosi, A., Waggoner, J.J., Babiker, A., Ahmed, R., Xie, X., et al. (2021). Infection and mRNA-1273 vaccine antibodies neutralize SARS-CoV-2 UK variant. medRxiv.

Greaney, A.J., Loes, A.N., Crawford, K.H.D., Starr, T.N., Malone, K.D., Chu, H.Y., and Bloom, J.D. (2021). Comprehensive mapping of mutations in the SARS-CoV-2 receptor-binding domain that affect recognition by polyclonal human plasma antibodies. Cell Host Microbe *29*, 463–476.e6.

Grehan, K., Ferrara, F., and Temperton, N. (2015). An optimised method for the production of MERS-CoV spike expressing viral pseudotypes. MethodsX *2*, 379–384.

Gu, Z., Eils, R., and Schlesner, M. (2016). Complex heatmaps reveal patterns and correlations in multidimensional genomic data. Bioinformatics *32*, 2847–2849.

Guthmiller, J.J., Stovicek, O., Wang, J., Changrob, S., Li, L., Halfmann, P., Zheng, N.Y., Utset, H., Stamper, C.T., Dugan, H.L., et al. (2021). SARS-CoV-2 Infection Severity Is Linked to Superior Humoral Immunity against the Spike. MBio *12*, e02940-20.

Howarth, M., Liu, W., Puthenveetil, S., Zheng, Y., Marshall, L.F., Schmidt, M.M., Wittrup, K.D., Bawendi, M.G., and Ting, A.Y. (2008). Monovalent, reduced-size quantum dots for imaging receptors on living cells. Nat. Methods *5*, 397–399.

Jiang, S., Hillyer, C., and Du, L. (2020). Neutralizing Antibodies against SARS-CoV-2 and Other Human Coronaviruses: (Trends in Immunology 41, 355-359; 2020). Trends Immunol. *41*, 545.

Kemp, S.A., Collier, D.A., Datir, R.P., Ferreira, I.A.T.M., Gayed, S., Jahun, A., Hosmillo, M., Rees-Spear, C., Mlcochova, P., Lumb, I.U., et al.; CITIID-NIHR BioResource COVID-19 Collaboration; COVID-19 Genomics UK (COG-UK) Consortium (2021). SARS-CoV-2 evolution during treatment of chronic infection. Nature.

Kim, S.I., Noh, J., Kim, S., Choi, Y., Yoo, D.K., Lee, Y., Lee, H., Jung, J., Kang, C.K., Song, K.H., et al. (2021). Stereotypic neutralizing V_H antibodies against SARS-CoV-2 spike protein receptor binding domain in patients with COVID-19 and healthy individuals. Sci. Transl. Med. *13*, eabd6990.

Laing, A.G., Lorenc, A., Del Molino Del Barrio, I., Das, A., Fish, M., Monin, L., Muñoz-Ruiz, M., McKenzie, D.R., Hayday, T.S., Francos-Quijorna, I., et al. (2020). A dynamic COVID-19 immune signature includes associations with poor prognosis. Nat. Med. *26*, 1623–1635.

Legros, V., Denolly, S., Vogrig, M., Boson, B., Siret, E., Rigaill, J., Pillet, S., Grattard, F., Gonzalo, S., Verhoeven, P., et al. (2021). A longitudinal study of SARS-CoV-2-infected patients reveals a high correlation between neutralizing antibodies and COVID-19 severity. Cell. Mol. Immunol. *18*, 318–327.

Li, Q., Wu, J., Nie, J., Zhang, L., Hao, H., Liu, S., Zhao, C., Zhang, Q., Liu, H., Nie, L., et al. (2020a). The Impact of Mutations in SARS-CoV-2 Spike on Viral Infectivity and Antigenicity. Cell *182*, 1284–1294.e1289.

Li, W., Chen, C., Drelich, A., Martinez, D.R., Gralinski, L.E., Sun, Z., Schäfer, A., Kulkarni, S.S., Liu, X., Leist, S.R., et al. (2020b). Rapid identification of a human antibody with high prophylactic and therapeutic efficacy in three animal models of SARS-CoV-2 infection. Proc. Natl. Acad. Sci. USA *117*, 29832–29838.

Liu, H., Wu, N.C., Yuan, M., Bangaru, S., Torres, J.L., Caniels, T.G., van Schooten, J., Zhu, X., Lee, C.D., Brouwer, P.J.M., et al. (2020a). Cross-Neutralization of a SARS-CoV-2 Antibody to a Functionally Conserved Site Is Mediated by Avidity. Immunity *53*, 1272–1280.e1275.

Liu, L., Wang, P., Nair, M.S., Yu, J., Rapp, M., Wang, Q., Luo, Y., Chan, J.F., Sahi, V., Figueroa, A., et al. (2020b). Potent neutralizing antibodies against multiple epitopes on SARS-CoV-2 spike. Nature *584*, 450–456.

Lu, R., Zhao, X., Li, J., Niu, P., Yang, B., Wu, H., Wang, W., Song, H., Huang, B., Zhu, N., et al. (2020). Genomic characterisation and epidemiology of 2019 novel coronavirus: implications for virus origins and receptor binding. Lancet *395*, 565–574.

McCallum, M., Marco, A., Lempp, F., Tortorici, M.A., Pinto, D., Walls, A.C., Beltramello, M., Chen, A., Liu, Z., Zatta, F., et al. (2021). N-terminal domain antigenic mapping reveals a site of vulnerability for SARS-CoV-2. Cell. https://doi.org/10.1016/j.cell.2021.03.028.

McCarthy, K.R., Rennick, L.J., Nambulli, S., Robinson-McCarthy, L.R., Bain, W.G., Haidar, G., and Duprex, W.P. (2021). Recurrent deletions in the SARS-CoV-2 spike glycoprotein drive antibody escape. Science *371*, 1139–1142.

Muecksch, F., Wise, H., Batchelor, B., Squires, M., Semple, E., Richardson, C., McGuire, J., Clearly, S., Furrie, E., Greig, N., et al. (2020). Longitudinal analysis of serology and neutralizing antibody levels in COVID19 convalescents. J. Infect. Dis.

Okba, N.M.A., Müller, M.A., Li, W., Wang, C., GeurtsvanKessel, C.H., Corman, V.M., Lamers, M.M., Sikkema, R.S., de Bruin, E., Chandler, F.D., et al. (2020). Severe Acute Respiratory Syndrome Coronavirus 2-Specific Antibody Responses in Coronavirus Disease Patients. Emerg. Infect. Dis. *26*, 1478–1488.

Piccoli, L., Park, Y.J., Tortorici, M.A., Czudnochowski, N., Walls, A.C., Beltramello, M., Silacci-Fregni, C., Pinto, D., Rosen, L.E., Bowen, J.E., et al. (2020). Mapping Neutralizing and Immunodominant Sites on the SARS-CoV-2 Spike Receptor-Binding Domain by Structure-Guided High-Resolution Serology. Cell *183*, 1024–1042.e1021.

Pickering, S., Betancor, G., Galão, R.P., Merrick, B., Signell, A.W., Wilson, H.D., Kia Ik, M.T., Seow, J., Graham, C., Acors, S., et al. (2020). Comparative assessment of multiple COVID-19 serological technologies supports continued evaluation of point-of-care lateral flow assays in hospital and community healthcare settings. PLoS Pathog. *16*, e1008817.

Pinto, D., Park, Y.J., Beltramello, M., Walls, A.C., Tortorici, M.A., Bianchi, S., Jaconi, S., Culap, K., Zatta, F., De Marco, A., et al. (2020). Cross-neutralization of SARS-CoV-2 by a human monoclonal SARS-CoV antibody. Nature *583*, 290–295.

Prévost, J., Gasser, R., Beaudoin-Bussières, G., Richard, J., Duerr, R., Laumaea, A., Anand, S.P., Goyette, G., Benlarbi, M., Ding, S., et al. (2020). Cross-Sectional Evaluation of Humoral Responses against SARS-CoV-2 Spike. Cell Rep Med *1*, 100126.

Rambaut, A., Loman, N., Pybus, O., Barclay, W., Barrett, J., Carabelli, A., Connor, T., Peacock, T., Robertson, D.L., Volz, E., et al. (2020). Preliminary genomic characterisation of an emergent SARS-CoV-2 lineage in the UK defined by a novel set of spike mutations.

Rees-Spear, C., Muir, L., Griffith, S.A., Heaney, J., Aldon, Y., Snitselaar, J.L., Thomas, P., Graham, C., Seow, J., Lee, N., et al.; SAFER Investigators (2021). The effect of spike mutations on SARS-CoV-2 neutralization. Cell Rep. *34*, 108890.

Robbiani, D.F., Gaebler, C., Muecksch, F., Lorenzi, J.C.C., Wang, Z., Cho, A., Agudelo, M., Barnes, C.O., Gazumyan, A., Finkin, S., et al. (2020). Convergent antibody responses to SARS-CoV-2 in convalescent individuals. Nature *584*, 437–442.

Rogers, T.F., Zhao, F., Huang, D., Beutler, N., Burns, A., He, W.T., Limbo, O., Smith, C., Song, G., Woehl, J., et al. (2020). Isolation of potent SARS-CoV-2 neutralizing antibodies and protection from disease in a small animal model. Science *369*, 956–963.

Rosa, A., Pye, V.E., Graham, C., Muir, L., Seow, J., Ng, K.W., Cook, N.J., Rees-Spear, C., Parker, E., Kassiotis, G., et al. (2021). SARS-CoV-2 recruits a haem metabolite to evade antibody immunity. medRxiv.

Scheid, J.F., Mouquet, H., Feldhahn, N., Seaman, M.S., Velinzon, K., Pietzsch, J., Ott, R.G., Anthony, R.M., Zebroski, H., Hurley, A., et al. (2009). Broad diversity of neutralizing antibodies isolated from memory B cells in HIV-infected individuals. Nature *458*, 636–640.

Seow, J., Graham, C., Merrick, B., Acors, S., Pickering, S., Steel, K.J.A., Hemmings, O., O'Byrne, A., Kouphou, N., Galao, R.P., et al. (2020). Longitudinal observation and decline of neutralizing antibody responses in the three months following SARS-CoV-2 infection in humans. Nat. Microbiol. *5*, 1598–1607.

Seydoux, E., Homad, L.J., MacCamy, A.J., Parks, K.R., Hurlburt, N.K., Jennewein, M.F., Akins, N.R., Stuart, A.B., Wan, Y.H., Feng, J., et al. (2020). Analysis of a SARS-CoV-2-Infected Individual Reveals Development of Potent Neutralizing Antibodies with Limited Somatic Mutation. Immunity *53*, 98–105.e105.

Shen, X., Tang, H., McDanal, C., Wagh, K., Fischer, W., Theiler, J., Yoon, H., Li, D., Haynes, B.F., Sanders, K.O., et al. (2021). SARS-CoV-2 variant B.1.1.7 is susceptible to neutralizing antibodies elicited by ancestral spike vaccines. Cell Host Microbe, S1931-3128(21)00102-5.

Starr, T.N., Greaney, A.J., Addetia, A., Hannon, W.W., Choudhary, M.C., Dingens, A.S., Li, J.Z., and Bloom, J.D. (2021). Prospective mapping of viral mutations that escape antibodies used to treat COVID-19. Science. https://doi.org/10.1126/science.abf9302.

Supasa, P., Zhou, D., Dejnirattisai, W., Liu, C., Mentzer, A.J., Ginn, H.M., Zhao, Y., Duyvesteyn, H.M.E., Nutalai, R., Tuekprakhon, A., et al. (2021). Reduced neutralization of SARS-CoV-2 B.1.1.7 variant by convalescent and vaccine sera. Cell. https://doi.org/10.1016/j.cell.2021.02.033.

ter Meulen, J., van den Brink, E.N., Poon, L.L., Marissen, W.E., Leung, C.S., Cox, F., Cheung, C.Y., Bakker, A.Q., Bogaards, J.A., van Deventer, E., et al. (2006). Human monoclonal antibody combination against SARS coronavirus: synergy and coverage of escape mutants. PLoS Med. *3*, e237.

Thompson, C.P., Grayson, N.E., Paton, R.S., Bolton, J.S., Lourenço, J., Penman, B.S., Lee, L.N., Odon, V., Mongkolsapaya, J., Chinnakannan, S., et al.; ISARIC4C Investigators (2020). Detection of neutralising antibodies to SARS-CoV-2 to determine population exposure in Scottish blood donors between March and May 2020. Euro Surveill. *25*, 2000685.

Tiller, T., Meffre, E., Yurasov, S., Tsuiji, M., Nussenzweig, M.C., and Wardemann, H. (2008). Efficient generation of monoclonal antibodies from single human B cells by single cell RT-PCR and expression vector cloning. J. Immunol. Methods *329*, 112–124.

Tortorici, M.A., Beltramello, M., Lempp, F.A., Pinto, D., Dang, H.V., Rosen, L.E., McCallum, M., Bowen, J., Minola, A., Jaconi, S., et al. (2020). Ultrapotent human antibodies protect against SARS-CoV-2 challenge via multiple mechanisms. Science *370*, 950–957.

van den Brink, E.N., Ter Meulen, J., Cox, F., Jongeneelen, M.A., Thijsse, A., Throsby, M., Marissen, W.E., Rood, P.M., Bakker, A.B., Gelderblom, H.R., et al. (2005). Molecular and biological characterization of human monoclonal antibodies binding to the spike and nucleocapsid proteins of severe acute respiratory syndrome coronavirus. J. Virol. *79*, 1635–1644.

von Boehmer, L., Liu, C., Ackerman, S., Gitlin, A.D., Wang, Q., Gazumyan, A., and Nussenzweig, M.C. (2016). Sequencing and cloning of antigen-specific antibodies from mouse memory B cells. Nat. Protoc. *11*, 1908–1923.

Walls, A.C., Park, Y.J., Tortorici, M.A., Wall, A., McGuire, A.T., and Veesler, D. (2020). Structure, Function, and Antigenicity of the SARS-CoV-2 Spike Glycoprotein. Cell *183*, 1735.

Wang, C., Li, W., Drabek, D., Okba, N.M.A., van Haperen, R., Osterhaus, A.D.M.E., van Kuppeveld, F.J.M., Haagmans, B.L., Grosveld, F., and Bosch, B.J. (2020). A human monoclonal antibody blocking SARS-CoV-2 infection. Nat. Commun. *11*, 2251.

Wang, P., Nair, M.S., Liu, L., Iketani, S., Luo, Y., Guo, Y., Wang, M., Yu, J., Zhang, B., Kwong, P.D., et al. (2021). Antibody Resistance of SARS-CoV-2 Variants B.1.351 and B.1.1.7. Nature. https://doi.org/10.1038/s41586-021-03398-2.

Watanabe, Y., Allen, J.D., Wrapp, D., McLellan, J.S., and Crispin, M. (2020). Site-specific glycan analysis of the SARS-CoV-2 spike. Science *369*, 330–333.

Weisblum, Y., Schmidt, F., Zhang, F., DaSilva, J., Poston, D., Lorenzi, J.C., Muecksch, F., Rutkowska, M., Hoffmann, H.H., Michailidis, E., et al. (2020). Escape from neutralizing antibodies by SARS-CoV-2 spike protein variants. eLife *9*, e61312.

Weissman, D., Alameh, M.G., de Silva, T., Collini, P., Hornsby, H., Brown, R., LaBranche, C.C., Edwards, R.J., Sutherland, L., Santra, S., et al. (2020). D614G Spike Mutation Increases SARS CoV-2 Susceptibility to Neutralization. Cell Host Microbe *29* (1), 23–31.e4, https://doi.org/10.1016/j.chom.2020.11.012.

Wibmer, C.K., Ayres, F., Hermanus, T., Madzivhandila, M., Kgagudi, P., Oosthuysen, B., Lambson, B.E., de Oliveira, T., Vermeulen, M., van der Berg, K., et al. (2021). SARS-CoV-2 501Y.V2 escapes neutralization by South African COVID-19 donor plasma. Nat. Med. https://doi.org/10.1038/s41591-021-01285-x.

Wickham, Hadley (2016). ggplot2: Elegant Graphics for Data Analysis (Springer-Verlag New York), 978-3-319-24277-4.

Winstone, H., Lista, M.J., Reid, A.C., Bouton, C., Pickering, S., Galao, R.P., Kerridge, C., Doores, K.J., Swanson, C., and Neil, S. (2021). The polybasic

CellPress
OPEN ACCESS

cleavage site in the SARS-CoV-2 spike modulates viral sensitivity to Type I interferon and IFITM2. J Virol. https://doi.org/10.1128/JVI.02422-20.

Wise, J. (2021). Covid-19: The E484K mutation and the risks it poses. BMJ *372*, n359.

Wrapp, D., Wang, N., Corbett, K.S., Goldsmith, J.A., Hsieh, C.L., Abiona, O., Graham, B.S., and McLellan, J.S. (2020). Cryo-EM structure of the 2019-nCoV spike in the prefusion conformation. Science *367*, 1260–1263.

Wu, N.C., Yuan, M., Liu, H., Lee, C.D., Zhu, X., Bangaru, S., Torres, J.L., Caniels, T.G., Brouwer, P.J.M., van Gils, M.J., et al. (2020a). An Alternative Binding Mode of IGHV3-53 Antibodies to the SARS-CoV-2 Receptor Binding Domain. Cell Rep. *33*, 108274.

Wu, Y., Wang, F., Shen, C., Peng, W., Li, D., Zhao, C., Li, Z., Li, S., Bi, Y., Yang, Y., et al. (2020b). A noncompeting pair of human neutralizing antibodies block COVID-19 virus binding to its receptor ACE2. Science *368*, 1274–1278.

Yang, Q., Hughes, T.A., Kelkar, A., Yu, X., Cheng, K., Park, S., Huang, W.C., Lovell, J.F., and Neelamegham, S. (2020). Inhibition of SARS-CoV-2 viral entry upon blocking N- and O-glycan elaboration. eLife *9*, e61552.

Yao, H., Song, Y., Chen, Y., Wu, N., Xu, J., Sun, C., Zhang, J., Weng, T., Zhang, Z., Wu, Z., et al. (2020). Molecular Architecture of the SARS-CoV-2 Virus. Cell *183*, 730–738.e713.

Yuan, M., Liu, H., Wu, N.C., Lee, C.D., Zhu, X., Zhao, F., Huang, D., Yu, W., Hua, Y., Tien, H., et al. (2020a). Structural basis of a shared antibody response to SARS-CoV-2. Science *369*, 1119–1123.

Yuan, M., Liu, H., Wu, N.C., and Wilson, I.A. (2020b). Recognition of the SARS-CoV-2 receptor binding domain by neutralizing antibodies. Biochem. Biophys. Res. Commun. https://doi.org/10.1016/j.bbrc.2020.10.012.

Yuan, M., Wu, N.C., Zhu, X., Lee, C.D., So, R.T.Y., Lv, H., Mok, C.K.P., and Wilson, I.A. (2020c). A highly conserved cryptic epitope in the receptor binding domains of SARS-CoV-2 and SARS-CoV. Science *368*, 630–633.

Yurkovetskiy, L., Wang, X., Pascal, K.E., Tomkins-Tinch, C., Nyalile, T.P., Wang, Y., Baum, A., Diehl, W.E., Dauphin, A., Carbone, C., et al. (2020). Structural and Functional Analysis of the D614G SARS-CoV-2 Spike Protein Variant. Cell *183*, 739–751.e738.

Zhou, T., Tsybovsky, Y., Gorman, J., Rapp, M., Cerutti, G., Chuang, G.Y., Katsamba, P.S., Sampson, J.M., Schon, A., Bimela, J., et al. (2020). Cryo-EM Structures of SARS-CoV-2 Spike without and with ACE2 Reveal a pH-Dependent Switch to Mediate Endosomal Positioning of Receptor-Binding Domains. Cell Host Microbe *28*, 867–879.e865.

Zhou, D., Dejnirattisai, W., Supasa, P., Liu, C., Mentzer, A.J., Ginn, H.M., Zhao, Y., Duyvesteyn, H.M.E., Tuekprakhon, A., Nutalai, R., et al. (2021). Evidence of escape of SARS-CoV-2 variant B.1.351 from natural and vaccine-induced sera. Cell, S0092-8674(21)00226-9.

Zohar, T., Loos, C., Fischinger, S., Atyeo, C., Wang, C., Slein, M.D., Burke, J., Yu, J., Feldman, J., Hauser, B.M., et al. (2020). Compromised Humoral Functional Evolution Tracks with SARS-CoV-2 Mortality. Cell *183*, 1508–1519 e1512.

Zost, S.J., Gilchuk, P., Case, J.B., Binshtein, E., Chen, R.E., Nkolola, J.P., Schäfer, A., Reidy, J.X., Trivette, A., Nargi, R.S., et al. (2020). Potently neutralizing and protective human antibodies against SARS-CoV-2. Nature *584*, 443–449.

Zufferey, R., Nagy, D., Mandel, R.J., Naldini, L., and Trono, D. (1997). Multiply attenuated lentiviral vector achieves efficient gene delivery in vivo. Nat. Biotechnol. *15*, 871–875.

STAR METHODS

KEY RESOURCES TABLE

REAGENT or RESOURCE	SOURCE	IDENTIFIER
Antibodies		
Goat-anti-human-IgM-HRP	Sigma	RRID: AB_258318<br>Cat#: A6907
Goat-anti-human-Fc-AP	Jackson	RRID: AB_2337608<br>Cat#:109-055-098
horse-anti-mouse-IgG-HRP	Cell Signaling Technology	Cat#: S7076
Mouse-anti-human IgG Fc-PE	Biolegend	RRID: AB_10895907<br>Cat#: 409304
anti-CD3-APC/Cy7	Biolegend	RRID: AB_10644011<br>Cat#: 344817
anti-CD8-APC-Cy7	Biolegend	RRID: AB_2044005<br>Cat#: 344713
anti-CD14-BV510	Biolegend	RRID: AB_2561379<br>Cat#: 301841
anti-CD19-PerCP-Cy5.5	Biolegend	RRID: AB_2275547<br>Cat#: 302229
anti-IgM-PE	Biolegend	RRID: AB_493006<br>Cat#: 314507
anti-IgD-Pacific Blue	Biolegend	RRID: AB_2561596<br>Cat#: 348223
anti-IgG-PeCy7	BD Biosciences	RRID: AB_10611712<br>Cat#: 561298
Srteptavidin-Alexa 488	Thermofisher Scientific	RRID: AB_2315383<br>Cat#: S32354
Streptavidin-APC	Thermofisher Scientific	Cat#: S32362
Streptavidin-PE	Thermofisher Scientific	Cat#: S21388
Murinized mAb CR3009	This manuscript (van den Brink et al., 2005)	N/A
mAb CR3022	This manuscript (ter Meulen et al., 2006)	N/A
SARS-CoV-2 specific nAbs and non-nAbs	This manuscript	N/A
"COVA" SARS-CoV-2 mAbs	Marit van Gils (Amsterdam) (Brouwer et al., 2020)	N/A
Bacterial and Virus Strains		
NEB® Stable Competent *E. coli*	New England Biolabs	Cat#: C3040H
SARS-CoV-2 Strain England 2 (England 02/2020/407073)	Public Health England (PHE)	N/A
Biological Samples		
PBMC and plasma from COVID-19 recovered individuals	(Laing et al., 2020)	N/A
Chemicals, Peptides, and Recombinant Proteins		
Polyethylenimine, Linear, MW 25000 (PEI Max)	Polysciences, Inc	Cat#: 23966
Polyethylenimine Hydrochloride, Linear, MW 4,000	Polysciences, Inc	Cat#: 24885
Recombinant S1	Peter Cherepanov (Crick) (Rosa et al., 2021)	N/A
Recombinant NTD	Peter Cherepanov (Crick) (Rosa et al., 2021)	N/A
Recombinant SARS-CoV-2 RBD	(Seow et al., 2020)	N/A

(*Continued on next page*)

Continued

REAGENT or RESOURCE	SOURCE	IDENTIFIER
Recombinant Stabilized SARS-CoV-2 Spike	Marit van Gils (Amsterdam) (Brouwer et al., 2020)	N/A
Recombinant SARS-CoV-2 Spike (biotinylated)	This manuscript	N/A
IdeS	Max Crispin (University of Southampton) (Dixon, 2014)	N/A
Recombinant S2 protein	SinoBiological	Cat#: 40590-V08B
Protein G agarose	GE Healthcare	Cat#: Cytiva 17-0618-02
HiTrap IMAC columns	GE Healthcare	Cat#: Cytiva 17-0921-04
HILOAD 16/600 SUPERDEX 200 PG	GE Healthcare	Cat#: 28989335
Strep-TactinXT Superflow 50% Suspension	IBA	Cat#: 2-4010-002
BioLock blocking solution	IBA	Cat#: 2-0205-050
Ni Sepharose® 6 Fast Flow	Cytiva	Cat#: GE17-5318-06
Bright-Glo Luciferase Assay System	Promega	Cat#: E2610
**Critical Commercial Assays**		
Q5® Site-Directed Mutagenesis Kit	New England Biolabs	Cat#: E0554
Bright-Glo luciferase kit	Promega	Cat#: E2610
QIAGEN Multiplex PCR kit	QIAGEN	Cat#: 206145
Phusion High-Fidelity DNA Polymerase	NEB	Cat#: E2611L
SuperScript III RT	Thermofisher Scientific	Cat#: 18080085
LIVE/DEAD Fixable Aqua Dead Cell Stain Kit	Thermofisher Scientific	Cat#: L34957
1-Step™ Ultra TMB-ELISA Substrate Solution	Thermofisher Scientific	Cat#: 34028
Phosphatase substrate	Sigma Aldrich	Cat#: S0942-200TAB
**Deposited Data**		
mAb sequence data	This manuscript	Accession numbers GenBank: MW802274 - MW802487
**Experimental Models: Cell Lines**		
FreeStyle™ 293F Cells	Thermofisher Scientific	Cat#: R79007
HEK293T/17	ATCC	ATCC® CRL-11268™
HeLa-ACE2	James Voss (Scripps), (Rogers et al., 2020)	N/A
Vero-E6	Wendy Barclay	ATCC® CRL-11268™
HEK293T	ATCC	ATCC® CRL-3216™
**Oligonucleotides**		
Heavy, kappa and Lambda PCR1 and 2 primers	(Scheid et al., 2009; Tiller et al., 2008; von Boehmer et al., 2016)	N/A
Spike mutagenesis primers	This manuscript	N/A
**Recombinant DNA**		
Biotinylated Spike (pHLSec)	This manuscript	N/A
Pre-fusion, stabilized and uncleaved SARS-CoV-2 Spike (pcDNA3.1+)	Marit van Gils (Amsterdam) (Brouwer et al., 2020)	N/A
Full length SARS-CoV-2 Spike (pcDNA3.1+)	Nigel Temperton (Seow et al., 2020)	N/A
Full length B.1.1.7 variant Spike (pcDNA3.1+)	Laura Mccoy (UCL) (Rees-Spear et al., 2021)	N/A
Full length SARS-CoV Spike (pcDNA3.1+)	This paper and (Winstone et al., 2021)	N/A
BirA	Addgene (Howarth et al., 2008)	Cat#: 20856
pHIV-Luc (constructed by replacing GFP in pHR'SIN-SEW (PMID: 11975847) with HA-luciferase)	Luis Apolonia (KCL)	N/A

(*Continued on next page*)

Continued

REAGENT or RESOURCE	SOURCE	IDENTIFIER
HIV 8.91 gag/pol packaging construct	p8.91 (Zufferey et al., 1997)	N/A
Heavy/Kappa/Lambda human IgG1 expression vectors	M. Nussenzweig (Rockefeller University) (von Boehmer et al., 2016)	N/A
Software and Algorithms		
FlowJo	Tree Star	https://www.flowjo.com
Prism	Graphpad	https://www.graphpad.com/scientific-software/prism/
Tableau	TABLEAU SOFTWARE, LLC	https://www.tableau.com/
IMGT/V-QUEST	IMGT	http://www.imgt.org/IMGT_vquest/vquest
R statistical programming environment	R Foundation for Statistical Computing	https://www.r-project.org
R studio	RStudio	https://www.rstudio.com/
ggplot2	(Wickham, 2016)	https://ggplot2.tidyverse.org
PyMol	The PyMOL Molecular Graphics System, Version 2.0 Schrödinger, LLC	https://www.pymol.org/
Other		
FACS Melody	BD Biosciences	N/A
Victor™ X3 multilabel reader	Perkin Elmer	N/A

RESOURCE AVAILABILITY

Lead contact

Further information and requests for resources and reagents should be directed to and will be fulfilled by the Lead Contact, Katie J Doores (katie.doores@kcl.ac.uk).

Materials availability

Reagents generated in this study are available from the Lead Contact with a completed Materials Transfer Agreement.

Data and Code availability

The antibody sequences generated during this study are available at GenBank (accession numbers Genbank: MW802274 - MW802487).

EXPERIMENTAL MODEL AND SUBJECT DETAILS

Human subjects

This study used human samples from three individuals collected as part of the COVID-IP study (Laing et al., 2020). All donors were male and P003, P008, P054 were 63, 29 and 62 years old, respectively. The study protocol for patient recruitment and sampling, out of the intensive care setting, was approved by the committee of the Infectious Diseases Biobank of King's College London with reference number COV-250320. The protocol for healthy volunteer recruitment and sampling was similarly approved by the same committee as an amendment to an existing approval for healthy volunteer recruitment with reference number MJ1-031218b. Both approvals were granted under the terms of the Infectious Disease Biobank's ethics permission (reference 19/SC/0232) granted by the South Central Hampshire B Research Ethics Committee in 2019. Patient recruitment from the ICU was undertaken through the ethics for the IMMERSE study approved by the South Central Berkshire Ethics Committee with reference number 19/SC/0187. Patient and control samples and data were anonymized at the point of sample collection by research nursing staff or clinicians involved in the COVID-IP project. We complied with all relevant ethical regulations.

Bacterial strains and cell culture

SARS-CoV-2 pseudotypes were produced by transfection of HEK293T/17 cells and neutralization activity assayed using HeLa cells stably expressing ACE2 (kind gift James E Voss). Small and large scale expression of monoclonal antibodies was performed in HEK293T/17 (ATCC; ATCC® CRL-11268) and 293 Freestyle cells (Thermofisher Scientific), respectively. Infectious virus was grown in Vero-E6 cells (kind gift from Wendy Barclay) and neutralization activity measured using the same cells. Full-length SARS-CoV Spike was expressed on the surface of HEK293T cells (ATCC® CRL-3216). Bacterial transformations were performed with NEB® Stable Competent *E. coli*.

Viruses

SARS-CoV-2 Strain England 2 (England 02/2020/407073) was obtained from Public Health England.

METHOD DETAILS

Protein expression and purification. Recombinant Spike and RBD for ELISA were expressed and purified as previously described (Seow et al., 2020). Recombinant S1 (residues 1-530) and NTD (residues 1-310) expression and purification was described in Rosa et al. (Rosa et al., 2021). S2 protein was obtained from SinoBiological (Cat number: 40590-V08B).

For antigen-specific B cell sorting, Spike glycoprotein consisted of the pre-fusion S ectodomain (residues 1–1138) with a GGGG substitution at the furin cleavage site (amino acids 682–685), proline substitutions at amino acid positions 986 and 987, and an N-terminal T4 trimerization domain. Spike was cloned into a pHLsec vector containing Avi and 6xHis tags (Aricescu et al., 2006). Biotinylated Spike was expressed in 1L of HEK293F cells (Thermofisher Scientific) at a density of 1.5×10^6 cells/mL. To achieve *in vivo* biotinylation, 480µg of Spike plasmid was co-transfected with 120µg of BirA (Howarth et al., 2008) and 12mg PEI-Max (1 mg/mL solution, Polysciences) in the presence of 200 µM biotin (final concentration). The supernatant was harvested after 7 days and purified using immobilized metal affinity chromatography and size-exclusion chromatography. Complete biotinylation was confirmed via depletion of protein using avidin beads.

Spike mutagenesis. SARS-CoV-2 mutants and the B.1.1.7 variant were previously reported (Rees-Spear et al., 2021). SARS-CoV-2 Spike D614G+ΔY144 (Forward primer CATAAGAACAACAAGAGC, Reverse primer ATAAACACCCAGGAAAGG) and B.1.1.7+E484K (Forward primer TAATGGCGTGAAGGGCTTCAATTGCTACTTC, Reverse primer CACGGTGTGCTGCCGGCC) were generated with Q5® Site-Directed Mutagenesis Kit (NEB, E0554) following the manufacturer's instructions.

ELISA (S, RBD, NTD, S2 or S1). 96-well plates (Corning, 3690) were coated with S, S1, NTD, S2 or RBD at 3 µg/mL overnight at 4°C. The plates were washed (5 times with PBS/0.05% Tween-20, PBS-T), blocked with blocking buffer (5% skimmed milk in PBS-T) for 1 h at room temperature. Serial dilutions of serum, plasma, mAb or supernatant in blocking buffer were added and incubated for 2 h at room temperature. Plates were washed (5 times with PBS-T) and secondary antibody was added and incubated for 1 h at room temperature. IgM was detected using Goat-anti-human-IgM-HRP (horseradish peroxidase) (1:1,000) (Sigma: A6907) and IgG was detected using Goat-anti-human-Fc-AP (alkaline phosphatase) (1:1,000) (Jackson: 109-055-098). Plates were washed (5 times with PBS-T) and developed with either AP substrate (Sigma) and read at 405 nm (AP) or 1-step TMB (3,3',5,5'-Tetramethylbenzidine) substrate (Thermo Scientific) and quenched with 0.5 M H_2S0_4 before reading at 450 nm (HRP).

Fab/Fc ELISA. 96-well plates (Corning, 3690) were coated with goat anti-human Fc IgG antibody at 3 µg/mL overnight at 4°C. The above protocol was followed. The presence of IgG in supernatants was detected using Goat-anti-human-Fc-AP (alkaline phosphatase) (1:1,000) (Jackson: 109-055-098).

IgG digestion to generate F(ab')$_2$. IgG were incubated with IdeS (Dixon, 2014) (4 µg of IdeS per 1 mg of IgG) in PBS for 1 h at 37°C. The Fc and IdeS were removed using a mix of Protein A Sepharose® Fast Flow (250 µL per 1 mg digested mAb; GE Healthcare Life Sciences) and Ni Sepharose 6 Fast Flow (50 µL per 1 mg digested mAb; GE Healthcare Life Sciences) which were washed twice with PBS before adding to the reaction mixture. After exactly 10 min the beads were removed from the F(ab')$_2$-dilution by filtration in Spin-X tube filters (Costar®) and the filtrate was concentrated in Amicon® Ultra Filters (10k, Millipore). Purified F(ab')$_2$ fragments were analyzed by SDS-PAGE.

F(ab')$_2$ and IgG competition ELISA. 96-well half area high bind microplates (Corning®) were coated with Spike protein at 3 µg/mL in PBS overnight at 4°C. Plates were washed (5 times with PBS/0.05% Tween-20, PBS-T) and blocked with 5% milk in PBS/T for 2 h at room temperature. Serial dilutions (5-fold) of F(ab')$_2$, starting at 100-molar excess of the IC_{80} of S binding, were added to the plates and incubated for 1 h at room temperature. Plates were washed (5 times with PBS-T) before competing IgG was added at their IC_{80} of S binding and incubated at room temperature for 1 h. Plates were washed (5 times with PBS-T) and Goat-anti-human-Fc-AP (alkaline phosphatase) (1:1,000) (Jackson: 109-055-098) was added and incubated for 45 min at room temperature. The plates were washed (5 times with PBS-T) and AP substrate (Sigma) was added. Optical density was measured at 405 nm in 5-min intervals. The percentage competition was calculated as the reduction in IgG binding in the presence of F(ab')$_2$ (at 100-molar excess of the IC_{80}) as a percentage of the maximum IgG binding in the absence of F(ab')$_2$. Competition groups were determined using Ward2 clustering (R, Complex Heatmap package (Gu et al., 2016)) for initial analysis and Groups were then arranged according to binding epitopes.

SARS-CoV-2 (wild-type and mutants) and SARS-CoV pseudotyped virus preparation. Pseudotyped HIV-1 virus incorporating the SARS-Cov-2 wild-type or mutants (D614G, N501Y, D614G+Δ69/70, D614G+ΔY144, B.1.1.7 and B.1.1.7+E484K) or SARS-CoV full-length Spike (Winstone et al., 2021) was produced in a 10 cm dish seeded the day prior with $5x10^6$ HEK293T/17 cells in 10 mL of complete Dulbecco's Modified Eagle's Medium (DMEM-C, 10% fetal bovine serum (FBS) and 1% Pen/Strep (100 IU/mL penicillin and 100 mg/mL streptomycin)). Cells were transfected using 90 µg of PEI-Max (1 mg/mL, Polysciences) with: 15 µg of HIV-luciferase plasmid, 10 µg of HIV 8.91 gag/pol plasmid (Zufferey et al., 1997) and 5 µg of SARS-CoV-2 spike protein plasmid (Grehan et al., 2015; Thompson et al., 2020). Pseudotyped virus was harvested after 72 h, filtered through a 0.45mm filter and stored at −80°C until required.

Neutralization assay with SARS-CoV-2 (wild-type and mutants) and SARS-CoV pseudotyped virus. Neutralization assays were conducted as previously described (Carter et al., 2020). Serial dilutions of serum samples (heat inactivated at 56°C for 30mins) or mAbs were prepared with DMEM-C media and incubated with pseudotyped virus for 1 h at 37°C in 96-well plates. Next, HeLa cells stably expressing the ACE2 receptor (provided by Dr James Voss, Scripps Research, La Jolla, CA) were added

(12,500 cells/50µL per well) and the plates were left for 72 h. The amount of infection was assessed in lysed cells with the Bright-Glo luciferase kit (Promega), using a Victor X3 multilabel reader (Perkin Elmer). Measurements were performed in duplicate and duplicates used to calculate the IC_{50} or ID_{50}.

Infectious virus strain and propagation. Vero-E6 cells (Cercopithecus aethiops derived epithelial kidney cells, provided by Prof Wendy Barclay, Imperial College London) cells were grown in Dulbecco's modified Eagle's medium (DMEM, GIBCO) supplemented with GlutaMAX, 10% fetal bovine serum (FBS), 20 µg/mL gentamicin, and incubated at 37°C with 5% CO_2. SARS-CoV-2 Strain England 2 (England 02/2020/407073) was obtained from Public Health England. The virus was propagated by infecting 60%–70% confluent Vero E6 cells in T75 flasks, at an MOI of 0.005 in 3 mL of DMEM supplemented with GlutaMAX and 10% FBS. Cells were incubated for 1 h at 37°C before adding 15 mL of the same medium. Supernatant was harvested 72 h post-infection following visible cytopathic effect (CPE), and filtered through a 0.22 µm filter, aliquoted and stored at −80C. The infectious virus titer was determined by plaque assay using Vero E6 cells.

Infectious virus neutralization assay. Vero-E6 cells were seeded at a concentration of 20,000 cells/100uL per well in 96-well plates and allowed to adhere overnight. Serial dilutions of mAbs were prepared with DMEM media (2% FBS and 1% Pen/Strep) and incubated with authentic SARS-CoV-2 for 1 h at 37°C. The media was removed from the pre-plated Vero-E6 cells and the serum-virus mixtures were added to the Vero E6 cells and incubated at 37°C for 24 h. The virus/serum mixture was aspirated, and each well was fixed with 150µL of 4% formalin at room temperature for 30 min and then topped up to 300µL using PBS. The cells were washed once with PBS and permeabilized with 0.1% Triton-X in PBS at room temperature for 15 min. The cells were washed twice with PBS and blocked using 3% milk in PBS at room temperature for 15 min. The blocking solution was removed and an N-specific mAb (murinized-CR3009 (van den Brink et al., 2005)) was added at 2 µg/mL (diluted using 1% milk in PBS) at room temperature for 45 min. The cells were washed twice with PBS and horse-anti-mouse-IgG-conjugated to HRP was added (1:2000 in 1% milk in PBS, Cell Signaling Technology, S7076) at room temperature for 45 min. The cells were washed twice with PBS, developed using TMB substrate for 30 min and quenched using 2M H_2SO_4 prior to reading at 450 nm. Measurements were performed in duplicate.

Antigen-specific B cell sorting. Fluorescence-activated cell sorting of cryopreserved PBMCs was performed on a BD FACS Melody. Sorting baits (SARS-CoV-2 Spike) were pre-complexed with the streptavidin fluorophore at a 1:4 molar ratio prior to addition to cells. PBMCs were stained with live/dead (fixable Aqua Dead, Thermofisher), anti-CD3-APC/Cy7 (Biolegend), anti-CD8-APC-Cy7 (Biolegend), anti-CD14-BV510 (Biolegend), anti-CD19-PerCP-Cy5.5 (Biolegend), anti-IgM-PE (Biolegend), anti-IgD-Pacific Blue (Biolegend) and anti-IgG-PeCy7 (BD) and Spike-Alexa488 (Thermofisher Scientific, S32354) and Spike-APC (Thermofisher Scientific, S32362). Live $CD3/CD8^{-}CD14^{-}CD19^{+}IgM^{-}IgD^{-}IgG^{+}Spike^{+}Spike^{+}$ cells were sorted into individual wells containing RNase OUT (Invitrogen), First Strand SuperScript III buffer, DTT and H_2O (Invitrogen) and RNA was converted into cDNA (SuperScript III Reverse Transcriptase, Invitrogen) using random hexamers (Bioline Reagents Ltd) following the manufacturer's protocol.

Full-length antibody cloning and expression. The human Ab variable regions of heavy and kappa/lambda chains were PCR amplified using previously described primers and PCR conditions (Scheid et al., 2009; Tiller et al., 2008; von Boehmer et al., 2016). PCR products were purified and cloned into human-IgG (Heavy, Kappa or Lambda) expression plasmids (von Boehmer et al., 2016) using the Gibson Assembly Master Mix (NEB) following the manufacturer's protocol. Gibson assembly products were directly transfected into HEK293T cells and transformed under ampicillin selection. Ab supernatants were harvested 3 days after transfection and IgG expression and Spike-reactivity determined using ELISA and concentrated 30-times for use in neutralization assays. Ab variable regions of heavy-light chain pairs that generated Spike reactive IgG were sequenced by Sanger sequencing.

IgG expression and purification. Ab heavy and light plasmids were co-transfected at a 1:1 ratio into HEK293F cells (Thermofisher) using PEI Max (1 mg/mL, Polysciences, Inc.) at a 3:1 ratio (PEI Max:DNA). Ab supernatants were harvested five days following transfection, filtered and purified using protein G affinity chromatography following the manufacturer's protocol (GE Healthcare).

Monoclonal antibody binding to Spike using flow cytometry. HEK293T cells were plated in a 6-well plate ($2x10^6$ cells/well). Cells were transfected with 1 µg of plasmid encoding full-length SARS-CoV or SARS-CoV-2 full-length Spike and incubated for 48 h at 37°C. Post incubation cells were resuspended in PBS and plated in 96-well plates ($1x10^5$ cells/well). Monoclonal antibodies were diluted in FACS buffer (1x PBS, 2% FBS, 1 mM EDTA) to 25 µg/mL and incubated with cells on ice for 1 h. The plates were washed twice in FACS buffer and stained with 50 µl/well of 1:200 dilution of PE-conjugated mouse anti-human IgG Fc antibody (BioLegend, 409304) on ice in dark for 1 h. After another two washes, stained cells were analyzed using flow cytometry, and the binding data were generated by calculating the percent (%) PE-positive cells using FlowJo 10 software.

ACE2 competition measured by flow cytometry. To prepare the fluorescent probe, 3.5 times molar excess of Streptavidin-PE (Thermofisher Scientific, S21388) was added to biotinylated SARS-CoV-2 Spike on ice. Additions were staggered over 5 steps with 30 min incubation times between each addition. Purified mAbs were mixed with PE conjugated SARS-CoV-2 S in a molar ratio of 4:1 in FACS buffer (2% FBS in PBS) on ice for 1 h. HeLa-ACE2 and HeLa cells were washed once with PBS and detached using PBS containing 5mM EDTA. Detached cells were washed and resuspended in FACS buffer. 0.5 million HeLa-ACE2 cells were added to each mAb-Spike complex and incubated on ice for 30 m. The cells were washed with PBS and resuspended in 200 µL FACS buffer with 1 µL of LIVE/DEAD Fixable Aqua Dead Cell Stain Kit (Invitrogen). HeLa and HeLa-ACE2 cells alone and with SARS-CoV-2 Spike only were used as background and positive controls, respectively. The geometric mean fluorescence for PE was measured from the gate of singlet and live cells. The ACE2 binding inhibition percentage was calculated with this equation: (Rogers et al., 2020)

$$\%\ ACE2\ binding\ inhibition = 100 * \left(1 - \frac{sample\ geometric\ mean - geomatric\ mean\ of\ background}{geometric\ mean\ of\ positive\ control - geometric\ mean\ of\ background}\right)$$

Monoclonal antibody sequence analysis. The heavy and light chain sequences of SARS-CoV-2 specific mAbs were examined using IMGT/V-QUEST(http://www.imgt.org/IMGT_vquest/vquest) to identify the germline usages, percentage of SHM and CDR region lengths. To remove variation introduced through cloning using mixture of forward primers, 5 amino acids or 15 nucleotides were trimmed from the start and end of the translated variable genes.

QUANTIFICATION AND STATISTICAL ANALYSIS

All neutralization and ELISA experiments were performed in duplicate. The 50% inhibitory concentrations/dilutions (IC/ID_{50}) or AUC (area under the cureve) were calculated using GraphPad Prism software. Statistical analysis in Figure 3 and Figure S2 (Mann-Whitney 2-sided U-test and Kruskal-Wallis multiple comparison test) were performed using GraphPad Prism software, significance defined as $p < 0.05$. Linear correlations (Figures 4 and S3, Spearman correlation) were also calculated using GraphPad Prism. Fold decrease in serum ID_{50} was calculated by dividing the average ID_{50} value for a given sample against SARS-CoV-2 or SARS-CoV-2 D614G (as indicated) by the average ID_{50} value for that sample against the indicated mutant or variant pseudovirus. In contrast, the fold decrease in mAb IC_{50} was calculated by dividing the average IC_{50} value for a given mAb against the indicated mutant pseudovirus by the average IC_{50} value for that mAb against the SARS-CoV-2 or SARS-CoV-2 D614G (as indicated).

Immunity, Volume *54*

Supplemental information

Neutralization potency of monoclonal antibodies recognizing dominant and subdominant epitopes on SARS-CoV-2 Spike is impacted by the B.1.1.7 variant

Carl Graham, Jeffrey Seow, Isabella Huettner, Hataf Khan, Neophytos Kouphou, Sam Acors, Helena Winstone, Suzanne Pickering, Rui Pedro Galao, Liane Dupont, Maria Jose Lista, Jose M. Jimenez-Guardeño, Adam G. Laing, Yin Wu, Magdalene Joseph, Luke Muir, Marit J. van Gils, Weng M. Ng, Helen M.E. Duyvesteyn, Yuguang Zhao, Thomas A. Bowden, Manu Shankar-Hari, Annachiara Rosa, Peter Cherepanov, Laura E. McCoy, Adrian C. Hayday, Stuart J.D. Neil, Michael H. Malim, and Katie J. Doores

Supplementary Figure S1: Donors used for B cell sorting have SARS-CoV-2 Spike reactive IgG+ B cells. Strategy to isolate SARS-CoV-2 Spike specific IgG$^+$ B cells. Example sorting for donor P008. Live CD3/CD8$^-$CD14$^-$CD19$^+$IgM$^-$IgD$^-$IgG$^+$Spike$^+$Spike$^+$ cells were sorted into individual wells. The heavy and light chains were reverse transcribed and amplified using nested PCR with gene specific primers. Related to **Figure 1**.

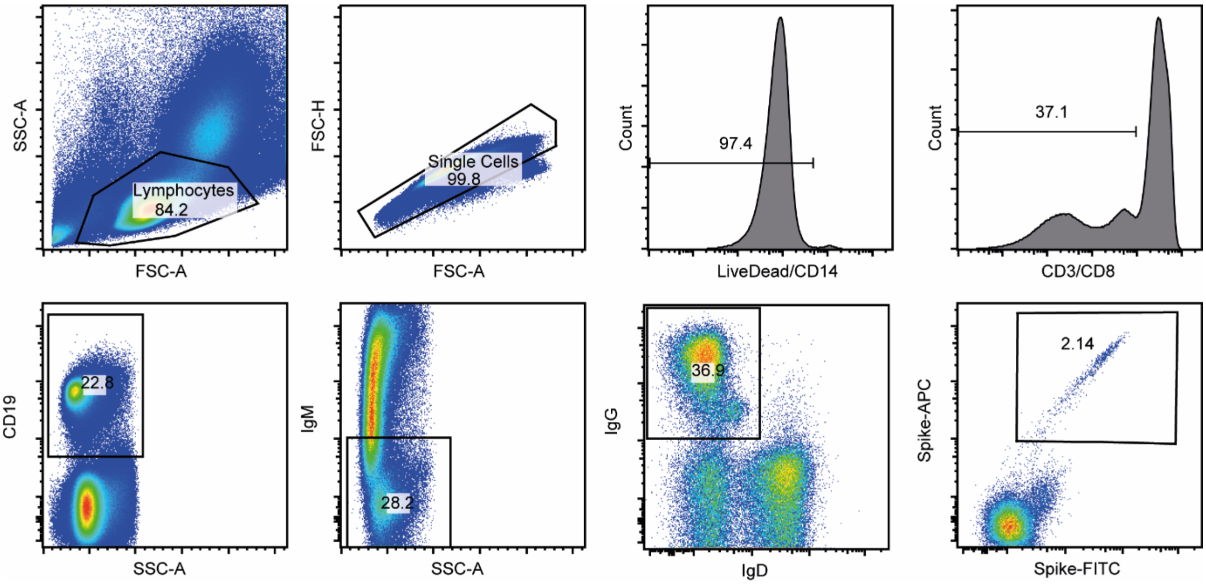

Supplementary Figure S2: SARS-CoV-2 specific mAbs have diverse gene usage. A) VH and VL germline gene usage for mAbs in the seven competition groups identified in **Figure 4A**. VH and VL genes are coloured according to the key. **B)** Pie charts showing percentage IGHV, IGHD, IGHJ, IGLV/IGLK and IGKJ/IGLJ usage for Spike reactive monoclonal antibodies. **C)** Bar graph showing the mean VH germline gene percentage usage in SARS-CoV-2 specific mAbs (blue) compared to a representative naïve repertoire (grey) (Briney et al., 2019). Error bars represent the standard deviation between donors used in the analysis (n = 3 for SARS-CoV-2 and n=10 for naïve repertoire). Differences between groups were determined using 2-way ANOVA with Šídák's multiple comparison test and *p* values <0.05 are shown. **D)** Single example of clonal expansion observed from P008. **E)** Sankey diagram showing the pairing between VH and VK or VL germline genes for SARS-CoV-2 mAbs. Related to **Figure 3**.

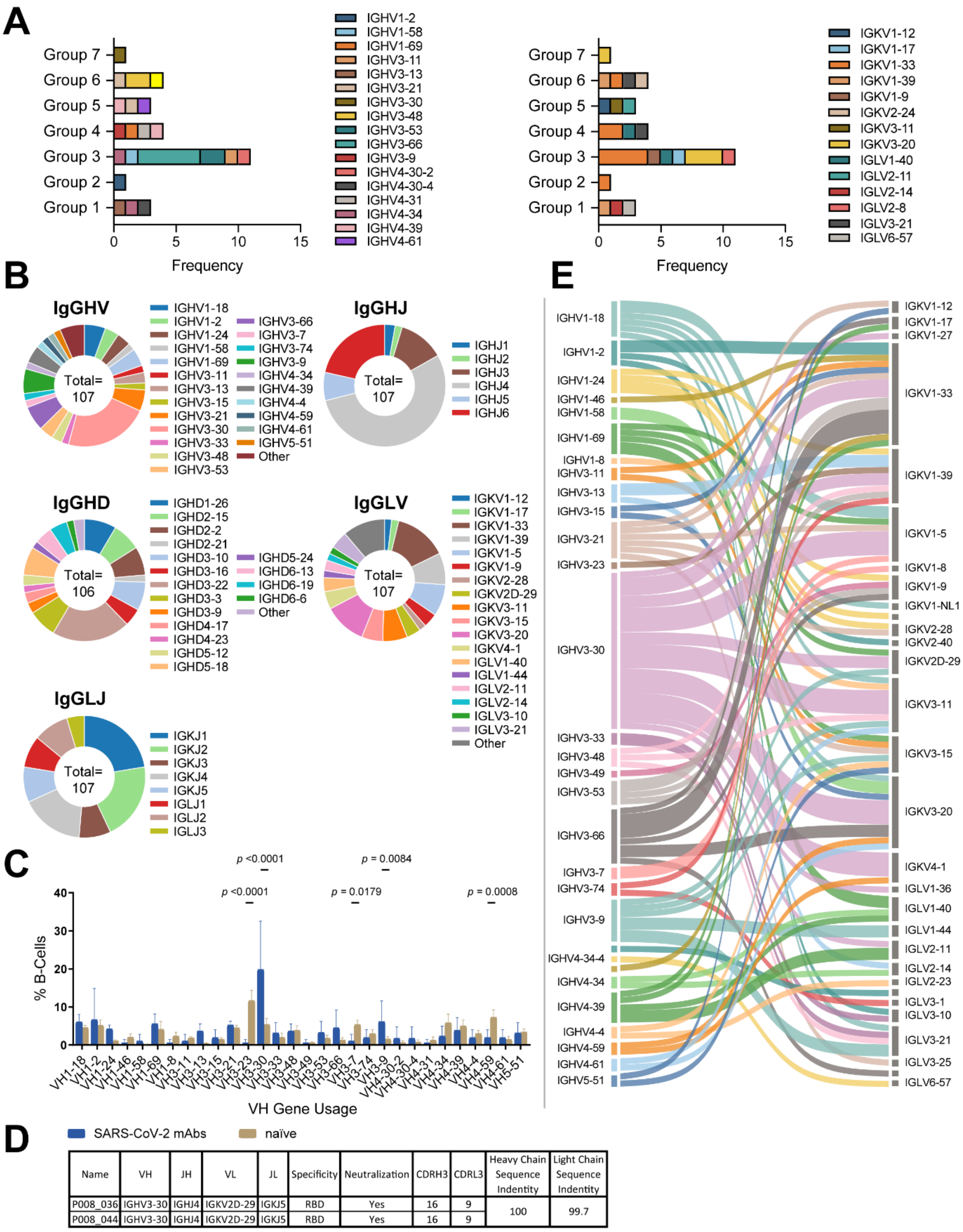

D

Name	VH	JH	VL	JL	Specificity	Neutralization	CDRH3	CDRL3	Heavy Chain Sequence Indentity	Light Chain Sequence Indentity
P008_036	IGHV3-30	IGHJ4	IGKV2D-29	IGKJ5	RBD	Yes	16	9	100	99.7
P008_044	IGHV3-30	IGHJ4	IGKV2D-29	IGKJ5	RBD	Yes	16	9		

Supplementary Figure S3: SARS-CoV-2 pseudovirus neutralization correlates and infectious virus neutralization and some nAbs cross-react with SARS-CoV. A) Correlation of mAb neutralization IC_{50} against infectious virus (y-axis) and pseudotyped virus (x-axis) (Spearman correlation, *r*. A linear regression was used to calculate the goodness of fit, r^2). **B)** Examples of shallow neutralization curves for Group 5 and Group 7 antibodies against SARS-CoV-2 pseudovirus. **C)** Group 6 nAbs show low neutralization plateaus against pseudovirus. **D)** Unlike the majority of other nAbs, Group 6 nAbs show 5-10 fold higher neutralization potency against infectious virus and reach neutralization plateaus of >95%. Binding of **E)** NTD and RBD nAbs and **F)** S2 non-neutralizing Abs to SARS-CoV-2 and SARS-CoV Spike proteins expressed on the surface of HEK 293T cells measured by flow cytometry. Also see, **Table S1**. Binding is reported as the % PE-positive cells. Antibodies are colour coded based on their competition group according to the key. **G)** Inhibition in binding of previously characterised SARS-CoV-2 nAbs (Brouwer et al., 2020) to Spike by $F(ab)_2$' fragments (generated through IdeS digestion of purified IgG) of representative members of each competition Group. Serial dilutions of $F(ab')_2$ (starting at 100-molar excess of the IC_{80} of Spike binding) were incubated for 1 hr before washing and addition of competing IgG (added at the IC_{80} of Spike binding). The percentage competition was calculated using the reduction in IgG binding in the presence of $F(ab')_2$ (at 100-molar excess of the IC_{80}) as a percentage of the maximum IgG binding in the absence of $F(ab')_2$. RBD, NTD and non-S1 specific nAbs were competed with the COVA mAbs specific for these domains only. Related **to Figure 4**.

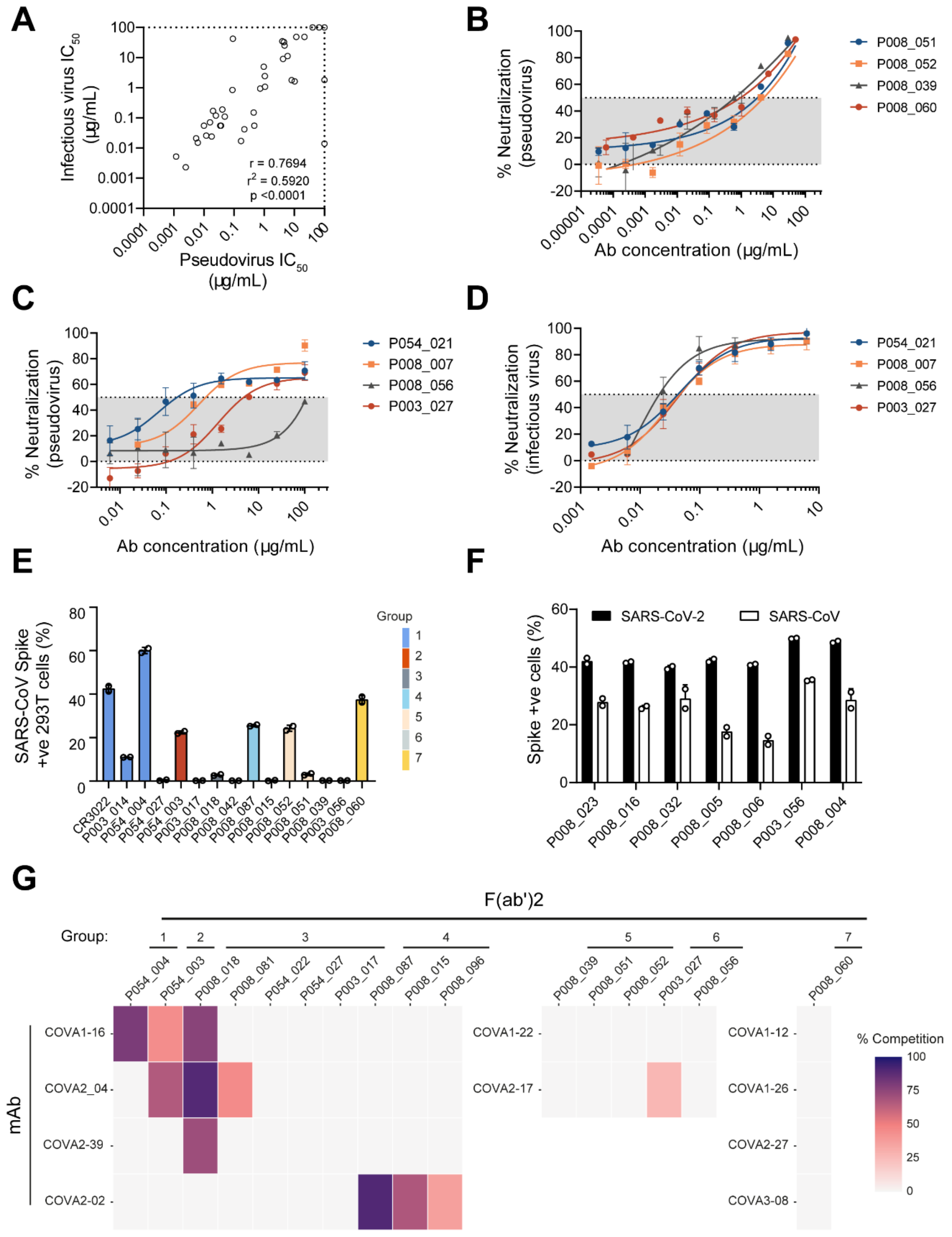

A
Infectious virus IC50 (µg/mL)
Pseudovirus IC50 (µg/mL)
r = 0.7694
r2 = 0.5920
p <0.0001
B
% Neutralization (pseudovirus)
Ab concentration (µg/mL)
P008_051
P008_052
P008_039
P008_060
C
% Neutralization (pseudovirus)
Ab concentration (µg/mL)
P054_021
P008_007
P008_056
P003_027
D
% Neutralization (infectious virus)
Ab concentration (µg/mL)
P054_021
P008_007
P008_056
P003_027
E
SARS-CoV Spike +ve 293T cells (%)
Group
CR3022
P003_014
P054_004
P054_027
P054_003
P003_017
P008_018
P008_042
P008_087
P008_015
P008_052
P008_051
P008_039
P003_056
P008_060
F
Spike +ve cells (%)
SARS-CoV-2
SARS-CoV
P008_023
P008_016
P008_032
P008_005
P008_006
P003_056
P008_004
G
F(ab')2
Group:
P054_004
P054_003
P008_018
P008_081
P054_022
P054_027
P003_017
P008_087
P008_015
P008_096
P008_039
P008_051
P008_052
P003_027
P008_056
P008_060
mAb
COVA1-16
COVA2_04
COVA2-39
COVA2-02
COVA1-22
COVA2-17
COVA1-12
COVA1-26
COVA2-27
COVA3-08
% Competition

Supplementary Figure S4: Some nAbs show reduced neutralization potency against B.1.1.7 and related Spike variants. Neutralization of mAbs and plasma were tested against pseudoviruses expressing variant SARS-CoV-2 Spikes including D614G, N501Y, D614G ΔH69/V70 and D614G ΔY144 mutations and the B.1.1.7 and B.1.1.7+E484K variants. **A)** Change in neutralization IC_{50} (μg/mL) for D614G and N501Y mutation compared to wild-type Spike, D614G+ΔH69/V70, D614G+ΔY144 compared to D614G Spike, and B.1.1.7 and B.1.1.7+E484K compared to D614G Spike. nAbs are coloured by competition group according to the key. **B)** Neutralization curves for all nAbs tested. Graphs are arranged by competition group and are colour coded according to the key. Related to **Figure 6**

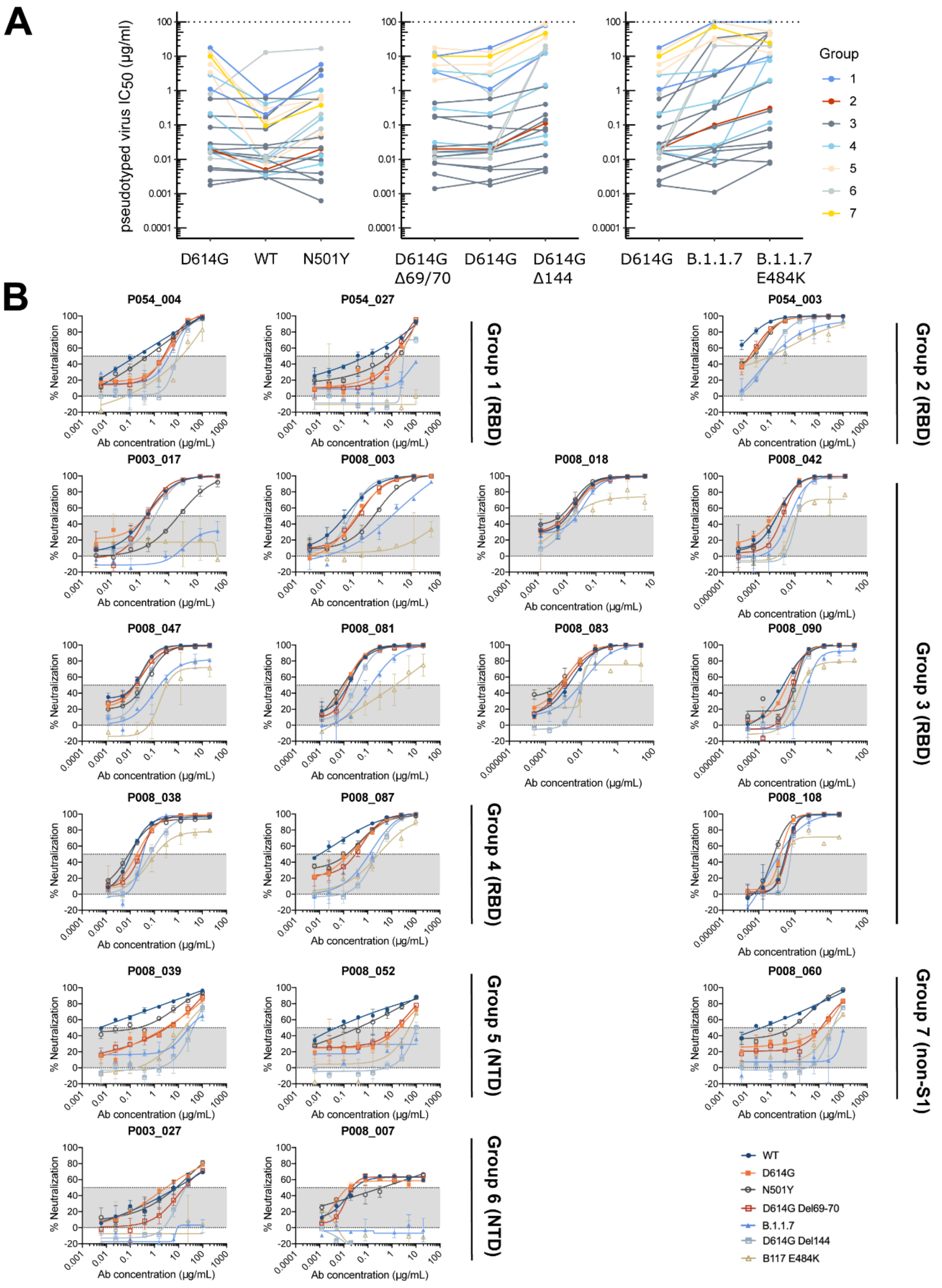
A
pseudotyped virus IC50 (µg/ml)
D614G
WT
N501Y
D614G Δ69/70
D614G
D614G Δ144
D614G
B.1.1.7
B.1.1.7 E484K
Group
1
2
3
4
5
6
7
B
P054_004
P054_027
Group 1 (RBD)
P054_003
Group 2 (RBD)
P003_017
P008_003
P008_018
P008_042
P008_047
P008_081
P008_083
P008_090
Group 3 (RBD)
P008_038
P008_087
Group 4 (RBD)
P008_108
P008_039
P008_052
Group 5 (NTD)
P008_060
Group 7 (non-S1)
P003_027
P008_007
Group 6 (NTD)
% Neutralization
Ab concentration (µg/mL)
WT
D614G
N501Y
D614G Del69-70
B.1.1.7
D614G Del144
B117 E484K

Figure S5: NTD-specific nAbs have the greatest reduction in neutralization potency against B.1.1.7 and related Spike variants. A) Fold changes in mAb IC_{50} compared to parent virus as indicated. **B)** Fold changes in plasma ID_{50} compared to parent virus as indicated. Each experiment was performed at least twice and the fold change is calculated from the average IC_{50} values. Related to **Figure 6** and **Figure S4**.

	Specificity	Group	From WT: D614G	From WT: N501Y	From D614G: D614G Del69/70	From D614G: D614G Del144	From D614G: B.1.1.7	From D614G: B.1.1.7 E484K	From B.1.1.7: B.1.1.7 E484K
P054_004	RBD	1	6	14	3	11	3	9	3
P054_027	RBD	1	25	8	-2	4	6	6	
P054_003	RBD	2	4	4	1	6	5	15	3
P003_017	RBD	3	1	25	-1	2	183	271	1
P008_003	RBD	3	1	9	2	-1	4	586	163
P008_018	RBD	3	1	-1	-1	5	1	4	3
P008_042	RBD	3	1	-1	1	2	3	4	1
P008_047	RBD	3	1	2	-1	7	3	9	3
P008_067	RBD	3	2	-5	-1	2	-2	2	4
P008_081	RBD	3	-1	-1	-1	7	18	102	6
P008_083	RBD	3	-1	-1	-2	2	4	4	-1
P008_090	RBD	3	1	3	2	1	4	6	1
P008_108	RBD	3	-2	-5	2	2	-2	4	7
P054_022	RBD	3	-1	-1	-1	2	5	87	18
P008_015	RBD	4	5	2	-1	2	-2	683	1166
P008_038	RBD	4	3	2	1	2	1	5	5
p008_087	RBD	4	22	15	1	7	2	9	4
P008_096	RBD	4	7	3	1	4	1	3	2
P008_039	NTD	5	450	7	-2	10	10	4	-3
P008_051	NTD	5	19	2	-1	15	5	7	1
P008_052	NTD	5	106	6	1	7	8	4	-2
P003_027	NTD	6	-16	1	16	20	125	125	
P008_007	NTD	6	1	17	-1	1216	1216	1216	
P054_021	NTD	6	1	8	1	1882	1882	1882	
P008_060	non-S1	7	105	4	1	5	7	2	-3

Specificity: RBD, NTD, non-S1

Group: 1, 2, 3, 4, 5, 6, 7

-fold loss neutralization; no difference; -fold increase neutralization; Base vius not neutralized

	From WT: D614G	From WT: N501Y	From D614G: D614G Del69/70	From D614G: D614G Del144	From D614G: B.1.1.7	From D614G: B.1.1.7 E484K	From B.1.1.7: B.1.1.7 E484K
P008	1	-1	1	2	8	n.d.	n.d.
P054	2	-2	-1	3	1	8	6

Table S1: SARS-CoV-2 mAbs target diverse epitopes. Gene usage, binding characteristics and neutralization properties of SARS-CoV-2 reactive mAbs expressed and purified on large scale. Antibodies are grouped by donor. Binding competition group for 27 mAbs is listed (see **Figure 4A**). EC_{50} was measured against Spike, RBD and NTD. For non-S1 binding mAbs, binding to S2 at 25 μg/mL was measured and + indicates binding. Neutralization ID_{50} was measured against infectious virus (with Vero-E6 target cells) and SARS-CoV-2 and SARS-CoV pseudovriuses (with HeLa-ACE2 target cells). The reported IC_{50} values are an average of three independent experiments. %ACE2 competition describes the ability of mAbs to prevent Spike binding to HeLa-ACE2 cells as measured by flow cytometry. The germline VH and VL usage is reported for each mAb. n.d., not determined. Related to **Figures 2, 3 and 4**.

mAb name	Specificty	Competition group	S EC50	RBD EC50	NTD EC50	S2 ELISA	SARS-CoV-2 WT FL IC50	SARS-CoV-2 WT PV IC50	SARS-CoV PV IC50	% ACE2 comp	VH	VL
P003_014	RBD	1	0.0076	0.0086	n.d.	n.d.	>100	>100	>50	86.3	IGHV3-13	IGKV1-39
P054_004	RBD	1	0.0360	0.0511	n.d.	n.d.	42.2300	0.2000	5.9585	95.2	IGHV4-34	IGLV2-14
P054_027	RBD	1	0.0077	0.0127	n.d.	n.d.	>100	0.7000	>50	92.9	IGHV4-30-4	IGLV6-57
P054_003	RBD	2	0.1756	0.2001	n.d.	n.d.	1.7900	0.0050	>100	98.5	IGHV1-2	IGKV1-33
P003_017	RBD	3	0.0057	0.0068	n.d.	n.d.	1.0800	0.1627	>100	96.5	IGHV3-66	IGKV1-9
P008_003	RBD	3	0.0756	0.0825	n.d.	n.d.	0.0170	0.0764	>50	99.7	IGHV4-34	IGLV1-40
P008_018	RBD	3	0.0714	0.0824	n.d.	n.d.	0.0550	0.0008	>50	99.9	IGHV3-66	IGKV3-20
P008_042	RBD	3	0.1277	0.1732	n.d.	n.d.	0.0240	0.0044	>50	99.9	IGHV3-66	IGKV3-20
P008_047	RBD	3	0.1731	0.2327	n.d.	n.d.	0.1893	0.0017	>50	99.7	IGHV3-53	IGKV1-33
P008_067	RBD	3	0.1638	0.1859	>20	n.d.	0.1700	0.0004	>50	99.1	IGHV3-66	IGKV1-17
P008_081	RBD	3	0.0384	0.0389	n.d.	n.d.	0.1159	0.0219	>50	99.5	IGHV1-58	IGKV3-20
P008_083	RBD	3	0.0203	0.1193	n.d.	n.d.	0.0260	0.0029	>50	99.9	IGHV3-66	IGKV1-33
P008_090	RBD	3	0.0182	0.0259	n.d.	n.d.	0.0709	0.0042	>50	99.9	IGHV3-53	IGKV1-33
P008_108	RBD	3	0.0092	0.0138	n.d.	n.d.	0.0023	0.0031	>50	100.0	IGHV3-11	IGKV1-33
P054_022	RBD	3	0.1741	0.1944	n.d.	n.d.	2.4400	0.5941	>50	98.5	IGHV4-30-2	IGLV2-8
P008_015	RBD	4	0.0337	0.0362	n.d.	n.d.	0.0553	0.0001	>50	80.2	IGHV3-9	IGLV3-21
P008_038	RBD	4	0.0578	0.0926	>10	n.d.	0.1100	0.0003	>50	42.3	IGHV1-69	IGLV1-40
P008_087	RBD	4	0.0212	0.0560	n.d.	n.d.	4.9600	0.0100	10.4600	48.5	IGHV4-31	IGKV1-33
P008_096	RBD	4	0.0299	0.0549	n.d.	n.d.	9.0920	0.4106	>50	50.6	IGHV4-39	IGKV1-33
P008_039	NTD	5	0.1014	>10	0.0240	n.d.	25.2800	0.0075	>50	91.2	IGHV3-21	IGKV1-12
P008_051	NTD	5	0.0914	>10	0.0561	n.d.	48.8200	0.3000	>50	74.5	IGHV4-61	IGKV3-11
P008_052	NTD	5	0.0853	>10	0.0625	n.d.	48.6500	0.1250	34.6400	80.2	IGHV4-39	IGLV2-11
P003_027	NTD	6	0.0039	>10	0.0065	n.d.	1.6400	12.9150	>50	47.4	IGHV3-48	IGKV1-39
P008_007	NTD	6	0.0700	>10	0.1710	n.d.	0.0549	0.0006	>100	66.3	IGHV3-48	IGLV3-21
P008_056	NTD	6	0.0602	>10	0.0488	n.d.	0.0136	>100	>50	36.8	IGHV3-21	IGKV1-33
P054_021	NTD	6	0.0263	>10	0.0643	n.d.	0.0421	0.0004	>100	51.0	IGHV1-24	IGKV2-24
P008_060	NON-S1	7	0.0311	>10	>10	n.d.	32.9000	0.0950	0.6434	50.7	IGHV3-30	IGKV3-20
P008_057	RBD	n.d.	0.0324	0.0597	>20	n.d.	0.0154	0.0063	>100	99.8	IGHV3-53	IGKV1-9
P008_070	RBD	n.d.	0.0342	0.1075	n.d.	n.d.	34.9500	4.0147	>100	47.3	IGHV3-30	IGKV1-33
P008_076	RBD	n.d.	0.0354	0.0384	n.d.	n.d.	0.8466	0.0889	0.4648	99.0	IGHV3-13	IGLV2-14
P008_086	RBD	n.d.	0.0338	0.0326	>20	n.d.	0.0052	0.0012	>100	99.3	IGHV1-58	IGKV3-20
P008_100	RBD	n.d.	0.0327	0.0364	>10	n.d.	11.3000	5.9766	>100	38.4	IGHV3-30	IGKV3-20
P008_103	RBD	n.d.	0.0663	0.1014	n.d.	n.d.	0.0215	0.0055	>100	99.8	IGHV3-66	IGKV1-33
P054_036	RBD	n.d.	0.0130	0.0293	>10	n.d.	0.0549	0.0100	>100	99.5	IGHV3-53	IGKV1-39
P054_050	RBD	n.d.	0.0268	0.0473	n.d.	n.d.	>100	66.6033	>100	60.1	IGHV3-13	IGKV1-39
P003_016	NTD	n.d.	0.1304	>20	2.0000	n.d.	0.1530	0.4494	>100	25.9	IGHV3-74	IGKV1-39
P003_055	NTD	n.d.	0.0600	>10	0.0546	n.d.	>100	>100	>100	n.d.	IGHV4-59	IGKV4-1
P008_001	NTD	n.d.	0.8157	>20	1.2000	n.d.	>100	>100	>100	37.3	IGHV3-49	IGKV1-9
P008_002	NTD	n.d.	0.0084	>20	0.0146	n.d.	>100	>100	>100	24.2	IGHV3-9	IGLV3-21
P008_014	NTD	n.d.	0.0662	>20	0.0215	n.d.	1.8120	>100	>100	43.0	IGHV3-15	IGKV1-33
P008_099	NTD	n.d.	0.0222	>20	0.1500	n.d.	>100	>100	>100	46.8	IGHV3-23	IGKV1-39
P054_044	NTD	n.d.	0.0308	>20	4.0000	n.d.	0.9428	0.7403	>100	46.7	IGHV1-69	IGKV1-5
P008_004	NON-S1	n.d.	0.0450	>10	>10	+	>100	>100	>100	61.4	IGHV3-7	IGKV1-5
P008_005	NON-S1	n.d.	0.2000	>10	>10	+	>100	>100	>100	22.8	IGHV3-30	IGKV1-5
P008_006	NON-S1	n.d.	0.1000	>10	>10	+	>100	>100	>100	16.0	IGHV3-30	IGKV1-5
P008_016	NON-S1	n.d.	0.1075	>10	>10	+	>100	>100	>100	39.9	IGHV4-59	IGKV3-20
P008_023	NON-S1	n.d.	0.4000	>10	>10	+	>100	>100	>100	22.7	IGHV3-11	IGKV3-15
P008_032	NON-S1	n.d.	0.0600	>10	>10	+	>100	>100	>100	23.3	IGHV3-30	IGKV3-11
